# The Clinicopathological Significance of the Cyclin D1/E1–Cyclin-Dependent Kinase (CDK2/4/6)–Retinoblastoma (RB1/pRB1) Pathway in Epithelial Ovarian Cancers

**DOI:** 10.3390/ijms25074060

**Published:** 2024-04-05

**Authors:** Ayat Lashen, Mashael Algethami, Shatha Alqahtani, Ahmed Shoqafi, Amera Sheha, Jennie N. Jeyapalan, Nigel P. Mongan, Emad A. Rakha, Srinivasan Madhusudan

**Affiliations:** 1 Naaz Coker Ovarian Cancer Research Centre, Nottingham Biodiscovery Institute, School of Medicine, University of Nottingham, University Park, Nottingham NG7 3RD, UK; mzxal1@exmail.nottingham.ac.uk (A.L.); mszma4@exmail.nottingham.ac.uk (M.A.); mzxsa20@exmail.nottingham.ac.uk (S.A.); msxas29@exmail.nottingham.ac.uk (A.S.); mzxas14@exmail.nottingham.ac.uk (A.S.); plzjnj@exmail.nottingham.ac.uk (J.N.J.); svznpm@exmail.nottingham.ac.uk (N.P.M.); mrzear1@exmail.nottingham.ac.uk (E.A.R.); 2 Department of Pathology, Nottingham University Hospital, City Campus, Nottingham NG5 1PB, UK; 3 Faculty of Medicine and Health Sciences, Centre for Cancer Sciences, University of Nottingham, Sutton Bonington Campus, Sutton Bonington LE12 5RD, UK; 4 Department of Pharmacology, Weill Cornell Medicine, New York, NY 10065, USA; 5 Department of Oncology, Nottingham University Hospitals, Nottingham NG5 1PB, UK

**Keywords:** cyclin D, cyclin E, CDK2, CDK4, CDK6, Rb, ovarian cancer, targeted therapy, prognostic biomarkers

## Abstract

Cyclin-dependent kinases (CDK2, CDK4, CDK6), cyclin D1, cyclin E1 and phosphorylated retinoblastoma (pRB1) are key regulators of the G1/S cell cycle checkpoint and may influence platinum response in ovarian cancers. CDK2/4/6 inhibitors are emerging targets in ovarian cancer therapeutics. In the current study, we evaluated the prognostic and predictive significance of the CDK2/4/6–cyclin D1/E1–pRB1 axis in clinical ovarian cancers (OC). The CDK2/4/6, cyclin D1/E1 and RB1/pRB1 protein expression were investigated in 300 ovarian cancers and correlated with clinicopathological parameters and patient outcomes. CDK2/4/6, cyclin D1/E1 and RB1 mRNA expression were evaluated in the publicly available ovarian TCGA dataset. We observed nuclear and cytoplasmic staining for CDK2/4/6, cyclins D1/E1 and RB1/pRB1 in OCs with varying percentages. Increased nuclear CDK2 and nuclear cyclin E1 expression was linked with poor progression-free survival (PFS) and a shorter overall survival (OS). Nuclear CDK6 was associated with poor OS. The cytoplasmic expression of CDK4, cyclin D1 and cyclin E1 also has predictive and/or prognostic significance in OCs. In the multivariate analysis, nuclear cyclin E1 was an independent predictor of poor PFS. Tumours with high nuclear cyclin E1/high nuclear CDK2 have a worse PFS and OS. Detailed bioinformatics in the TCGA cohort showed a positive correlation between cyclin E1 and CDK2. We also showed that cyclin-E1-overexpressing tumours are enriched for genes involved in insulin signalling and release. Our data not only identified the prognostic/predictive significance of these key cell cycle regulators but also demonstrate the importance of sub-cellular localisation. CDK2 targeting in cyclin-E1-amplified OCs could be a rational approach.

## 1. Introduction

Ovarian cancer is the most common cause of gynaecological cancer deaths [1]. Recent advances in surgery, systemic chemotherapy, and targeted therapy have improved the clinical outcomes [2,3,4,5]. However, intrinsic and acquired resistance to the current therapies pose considerable clinical challenges [2,3,4,5]. Therefore, the development of new biomarkers and therapeutic targets remains an area of high priority in ovarian cancer. 

In normal cells, including ovarian epithelial cells, the cell cycle progression includes four well defined phases [6,7,8,9]. During the G1 phase, the cells synthesise essential proteins, transcripts and organelles needed for DNA synthesis. The cells then progress to the S phase, where DNA replication occurs. This is followed by the G2 phase, where microtubules are assembled, and finally, during the M phase, cell division is completed. When the cells are fully differentiated or deprived of mitogenic signals, they enter into a quiescent G0 phase. Cell cycle progression is highly regulated. During G0, the transcriptional activity of the E2F transcription factor is repressed by retinoblastoma protein (Rb). Following mitogenic stimulation, the cells enter G1, requiring CDK3−cyclin C, which phosphorylates Rb at Ser807/811. During early G1, the D-type cyclins (e.g., cyclin D1) bind and activate CDK4 and/or CDK6. CDK4/6-cyclin D1 partially phosphorylates Rb, which activate E2F. Although E2F is still bound to Rb, it can transcribe several genes, including CCNE1 (cyclin E1), CCNA2, CCNB1, CDK2 and CDK1. During late G1, the binding of cyclin E to CDK2 results in the further phosphorylation of Rb, which releases and fully activates E2F. Following the transcription of S-phase proteins, directed by E2F, the cells then move to the S phase of the cell cycle. The subsequent sustained phosphorylation of Rb by CDK2−cyclin A facilitates the S/G2 transition, with CDK1−cyclin A allowing the commencement of mitosis and with CDK1−cyclin B ensuring progression through the M phase. Finally, the degradation of cyclin B and the dephosphorylation of Rb (by the PP1 and PP2A phosphatases) return the cells to the G1 phase of the cell cycle [6,7,8,9]. 

In epithelial ovarian cancer cells, the cell cycle’s regulatory processes are highly dysregulated, resulting in replication stress, genomic instability, and unregulated cell proliferation [10,11]. Aberrations in the cyclin D1/E1–cyclin-dependent kinase (CDK2/4/6)–retinoblastoma (RB) pathway can promote an aggressive ovarian cancer phenotype. In addition, the dysregulation of cyclins–CDKs may also contribute to platinum resistance. In ovarian cancer cells, platinum treatment can phosphorylate CDK6, which has been shown to stabilise the FOXO3 transcription factor, resulting in the upregulation of ATR, a key DNA repair factor involved in platinum resistance [12]. CDK4/6 inhibitors such as palbociclib, abemaciclib and ribociclib have transformed the lives of patients with ER+/HER2- advanced breast cancers [13,14,15,16]. In addition, abemaciclib and ribociclib can also improve the survival outcomes in high-risk, ER+/HER2-, early-stage breast cancers [17,18]. Whether a similar clinical benefit can be realised in epithelial ovarian cancer remains to be established. 

In the current study, we hypothesised that the cyclin D1/E1–CDK2/4/6–RB pathway may influence ovarian cancer prognosis and predict therapy resistance. To address this hypothesis, we have comprehensively evaluated the clinicopathological significance of the CDK2, CDK4, CDK6, cyclin D1, cyclin E1, RB1 and pRB1(Ser 795) protein expression in a clinical cohort of epithelial ovarian cancer. The *CDK2*, *CDK4*, *CDK6*, cyclin D1, cyclin E1 and *RB1* transcript expression was evaluated in publicly available datasets.

## 2. Results

### 2.1. CDK2 Expression 

**Figure 1:**
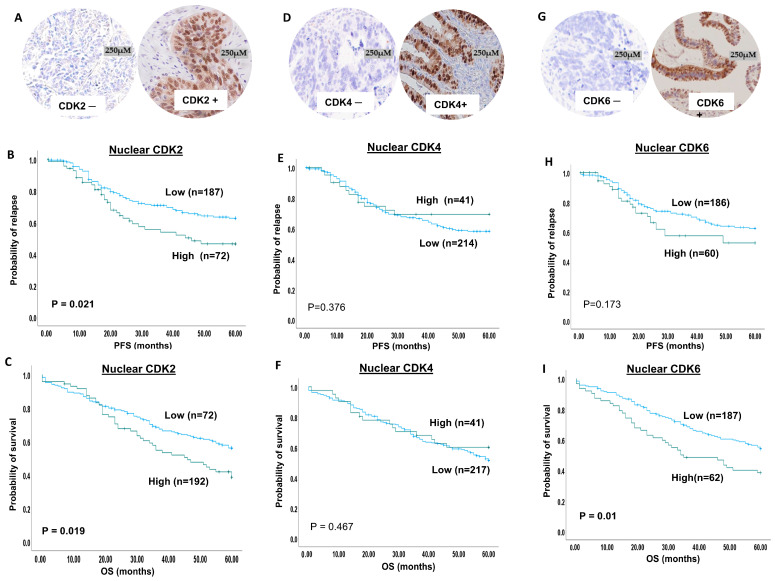
**CDK2, 4 and 6 immunohistochemical expression in ovarian cancers.** (**A**) CDK2 protein expression in ovarian cancer (panel on the left side of figure shows negative CDK2 expression, and the panel on the right side shows moderate to strong CDK2 expression). (**B**) Kaplan–Meier curve for CDK2 nuclear expression and progression-free survival (blue line = low expression, green line = high expression). (**C**) Kaplan–Meier curve for CDK2 nuclear expression and overall survival (blue line = low expression, green line = high expression). (**D**) CDK4 protein expression in ovarian cancer (panel on the left side of figure shows negative CDK4 expression, and the panel on the right side shows moderate to strong CDK4 expression). (**E**) Kaplan–Meier curve for CDK4 nuclear expression and progression-free survival (blue line = low expression, green line = high expression). (**F**) Kaplan–Meier curve for CDK4 nuclear expression and overall survival (blue line = low expression, green line = high expression). (**G**) CDK6 protein expression in ovarian cancer (panel on the left side of figure shows negative CDK6 expression, and the panel on the right side shows moderate to strong CDK6 expression). (**H**) Kaplan–Meier curve for CDK6 nuclear expression and progression-free survival (blue line = low expression, green line = high expression). (**I**) Kaplan–Meier curve for CDK6 nuclear expression and overall survival (blue line = low expression, green line = high expression).

We observed both nuclear and cytoplasmic immunohistochemical staining for CDK2 expression. High nuclear CDK2 was seen in 28% and high cytoplasmic CDK2 was observed in 28% of ovarian cancer (Figure 1A). 

High nuclear CDK2 expression was associated with high-grade disease (adjusted *p* value = 0.005, Table 1). High cytoplasmic CDK2 expression showed a significant association with serous carcinoma (adjusted *p* value = 0.005, Supplementary Table S2). Tumours showing a combined high nuclear and high cytoplasmic CDK2 expression were significantly linked with serous cancer (adjusted *p* value = 0.007, Supplementary Table S3) and a high grade (adjusted *p* value = 0.02, Supplementary Table S3). 

The outcome analysis showed a significant association between high nuclear CDK2 and a shorter progression-free survival (PFS) (*p* = 0.021) (Figure 1B) and a shorter overall survival (OS) (*p* = 0.019) (Figure 1C). However, neither cytoplasmic expression (Supplementary Figure S2A,C) nor combined nuclear and cytoplasmic co-expression influenced the patient outcome (Supplementary Figure S2B,D). 

At the transcriptomic level, we observed a significantly higher expression of *CDK2* in the ovarian tumour tissues compared to the normal tissues (Supplementary Figure S2E). However, there was no significant association between the overall expression of *CDK2* and the outcome in terms of PFS or OS (Supplementary Figure S2F,G).

### 2.2. CDK4 Expression 

The expression of CDK4 was dichotomised into high and low expression based on X-tile software, as described in the Section 5. High nuclear CDK4 was observed in 41/255 (16%) and high cytoplasmic CDK4 was observed in 77/255 (31%) of tumours (Figure 1D). 

Although the nuclear expression of CDK4 did not show an association with the clinicopathological parameters (Supplementary Table S4), high cytoplasmic expression of CDK4 showed a significant association with serous carcinoma (adjusted *p* value < 0.0001), a high tumour grade (adjusted *p* value < 0.0001), stage 3 tumours (adjusted *p* = 0.003) and residual tumours after surgery (adjusted *p* value =0.036) (Supplementary Table S5). High nuclear and high cytoplasmic CDK4 co-expression was significantly linked with serous cancer (adjusted *p* = 0.01) and a high grade (adjusted *p* = 0.007) (Supplementary Table S6).

Similarly, the survival analysis revealed no association between nuclear CDK4 and patient outcome (Figure 1E,F). However, high cytoplasmic expression of CDK4 was significantly associated with a poor outcome in terms of a shorter PFS (*p* < 0.0001) (Supplementary Figure S3A) and a shorter OS (*p* = 0.017) (Supplementary Figure S3C). High cytoplasmic/low nuclear co-expression showed a significant association with a shorter PFS (*p* = 0.008) (Supplementary Figure S3B) but not with OS (Supplementary Figure S3D).

At the transcriptomic level, we observed a significantly higher expression of *CDK4* in the ovarian tumour tissues compared to the normal tissues (Supplementary Figure S3E). However, *CDK4* mRNA expression did not influence survival (Supplementary Figure S3F,G).

### 2.3. CDK6 Expression

We observed nuclear and cytoplasmic staining for CDK4 (Figure 1G). Low CDK6 nuclear expression was observed in 74% (209/281) of patients, while high CDK6 nuclear expression was observed in 26% (72/281) of patients. Low CDK6 cytoplasmic expression was observed in 91% (257/281) of patients, while high CDK6 cytoplasmic expression was observed in 9% (24/281) of patients.

Neither nuclear CDK6, cytoplasmic CDK6, nor their co-expression was associated with any aggressive parameters (Supplementary Tables S7–S9). 

CDK6 nuclear expression did not influence PFS (*p* = 0.17) (Figure 1H), but high nuclear CDK6 was significantly associated with poor OS (*p* = 0.01) (Figure 1I). Cytoplasmic CDK6 expression did not influence survival (Supplementary Figures S3C and S4A). Nuclear and cytoplasmic CDK6 co-expression did not influence PFS (Supplementary Figure S4B), but high nuclear and cytoplasmic CDK6 expression was linked with poor OS (*p* = 0.027) (Supplementary Figure S4D).

At the transcriptomic level, there was a significant difference in expression between the normal and tumour ovarian tissues (Supplementary Figure S4E). However, no association was observed in terms of survival (Supplementary Figure S4F,G).

### 2.4. Cyclin D1 Expression in Ovarian Cancer

**Figure 2:**
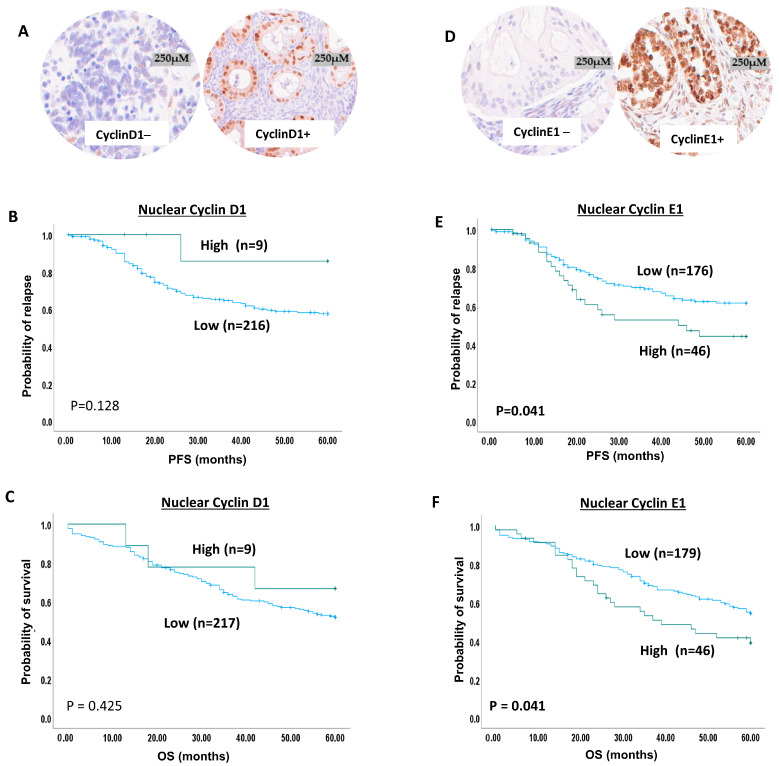
**Cyclin E1 and D1 immunohistochemical expression in ovarian cancers.** (**A**) Cyclin D1 protein expression in ovarian cancer (panel on the left side of figure shows negative cyclin D1 expression, and the panel on the right side shows moderate to strong cyclin D1 expression). (**B**) Kaplan–Meier curve for cyclin D1 nuclear expression and progression-free survival (blue line = low expression, green line = high expression). (**C**) Kaplan–Meier curve for cyclin D1 nuclear expression and overall survival (blue line = low expression, green line = high expression). (**D**) Cyclin E1 protein expression in ovarian cancer (panel on the left side of figure shows negative cyclin E1 expression, and the panel on the right side shows moderate to strong cyclin E1 expression). (**E**) Kaplan–Meier curve for cyclin E1 nuclear expression and progression-free survival (blue line = low expression, green line = high expression). (**F**) Kaplan–Meier curve for cyclin E1 nuclear expression and overall survival (blue line = low expression, green line = high expression).

Cyclin D1 staining was observed in the nucleus and in the cytoplasm (Figure 2A). Low cyclin D1 nuclear expression was found in 96% (247/256) of patients, while high cyclin D1 nuclear expression was observed in 4% (9/256) of patients. Low Cyclin D1 cytoplasmic expression was found in 91% (234/256) of patients, while high cyclin D1 cytoplasmic expression was observed in 9% (22/256) of patients.

Low nuclear cyclin D1 showed a significant association with serous carcinoma (adjusted *p* value = 0.005) and a high tumour grade (adjusted *p* value < 0.0001) (Supplementary Table S10). However, the cytoplasmic expression of cyclin D1 did not show an association with the pathological parameters (Supplementary Table S11). Low nuclear and low cytoplasmic cyclin D1 co-expression showed a significant association with a high tumour grade (adjusted *p* value = 0.035) (Supplementary Table S12).

Nuclear cyclin D1 was not associated with survival (Figure 2B,C); nor was low cytoplasmic cyclin D1 expression associated with PFS (*p* = 0.62) (Supplementary Figure S5A), but it was significantly associated with a shorter OS (*p* = 0.028) (Supplementary Figure S5C). Nuclear and cytoplasmic cyclin D1 co-expression did not influence the patient outcome (Supplementary Figure S5B,D).

There was a statistically significant higher gene expression of *CCND1* in the ovarian tumours compared to the normal tissues (Supplementary Figure S5E). However, there was no association between *CCND1* mRNA expression and patient survival (Supplementary Figure S5F,G).

### 2.5. Cyclin E1 Protein Expression in Ovarian Cancer

We observed nuclear and cytoplasmic staining for cyclin E1 (Figure 2D). A total of 80% (207/260) of patients showed low cyclin E1 nuclear expression, while 20% (53/260) of patients had high cyclin E1 nuclear expression. Low cyclin E1 cytoplasmic expression was found in 30% (79/260) of patients, while high cyclin E1 cytoplasmic expression was observed in 70% (181/260) of patients.

No significant clinicopathological associations were observed for nuclear cyclin E1 expression (Supplementary Table S13). High cytoplasmic cyclin E1 expression showed a significant association with serous carcinoma (adjusted *p* value < 0.0001) (Supplementary Table S14). High nuclear and cytoplasmic co-expression was also significantly associated with serous carcinoma (adjusted *p* value = 0.006) (Supplementary Table S15).

High nuclear cyclin E1 showed a significant association with a shorter PFS (*p* = 0.041) (Figure 2E) and poor OS (*p* = 0.041) (Figure 2F). Similarly, high cytoplasmic expression was significantly associated with a shorter PFS (*p* = 0.047) (Supplementary Figure S6A) and a shorter OS (*p* = 0.015) (Supplementary Figure S6C). Moreover, high nuclear and cytoplasmic co-expression had a significant association with a short PFS (*p* = 0.021) (Supplementary Figure S6B) and a shorter OS (*p* = 0.006) (Supplementary Figure S6D). 

A higher expression of *CCNE1* mRNA was observed in the ovarian tumours compared to the normal tissue (Supplementary Figure S5E). High *CCNE1* transcripts were significantly associated with poor PFS (*p* = 0.018) (Supplementary Figure S6F) and poor OS (*p* = 0.0009) (Supplementary Figure S6G).

### 2.6. RB1 Expression and Ovarian Cancer

**Figure 3:**
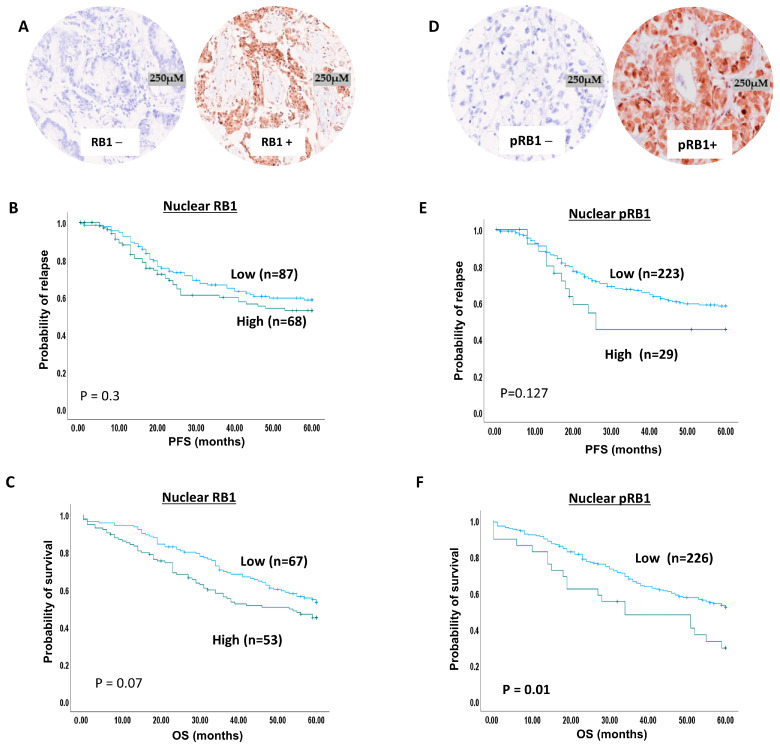
**RB1 and pRB1 immunohistochemical expression in ovarian cancers.** (**A**) RB1 protein expression in ovarian cancer (panel on the left side of figure shows negative RB1 expression, and the panel on the right side shows moderate to strong RB1 expression). (**B**) Kaplan–Meier curve for RB1 nuclear expression and progression-free survival (blue line = low expression, green line = high expression). (**C**) Kaplan–Meier curve for RB1 nuclear expression and overall survival (blue line = low expression, green line = high expression). (**D**) pRB1 protein expression in ovarian cancer (panel on the left side of figure shows negative pRB1 expression, and the panel on the right side shows moderate to strong pRB1 expression). (**E**) Kaplan–Meier curve for pRB1 nuclear expression and progression-free survival (blue line = low expression, green line = high expression). (**F**) Kaplan–Meier curve for pRB1 nuclear expression and overall survival (blue line = low expression, green line = high expression).

RB1 staining was observed in the nucleus and in the cytoplasm (Figure 3A). Low RB1 nuclear expression was found in 55% (161/292) of patients, while high RB1 nuclear expression was observed in 45% (131/292) of patients. Low RB1 cytoplasmic expression was found in 88% (256/292) of patients, while high RB1 cytoplasmic expression was observed in 12% (36/292) of patients.

No significant associations were observed for nuclear RB1, cytoplasmic RB1 or nuclear/cytoplasmic co-expression (Supplementary Table S16, Supplementary Table S17 and Supplementary Table S18, respectively). 

Nuclear RB1 did not influence survival (Figure 3B,C). Cytoplasmic RB1 did not influence PFS or OS (Supplementary Figure S7A,C). Nuclear/cytoplasmic co-expression also did not influence survival (Supplementary Figure S7B,D). 

A lower expression of *RB1* mRNA was found in the ovarian tumours compared to normal tissue (*p* = 0.01) (Supplementary Figure S7E). In contrast, high *RB1* mRNA expression levels were associated with poor PFS (*p* = 0.0003) (Supplementary Figure S7F) and poor OS (*p* = 0.0005) (Supplementary Figure S7G).

### 2.7. pRb1 Expression and Ovarian Cancer

CDK4 is essential for the phosphorylation of RB1 at Ser795 [19]. We observed nuclear and cytoplasmic staining for pRB1 (Figure 3D). A total of 94% (257/292) of patients showed low pRB1 nuclear expression, while 6% (35/292) of patients had high pRB1 nuclear expression. Low pRB1 cytoplasmic expression was found in 87% (253/292) of patients, while high pRB1 cytoplasmic expression was observed in 13% (39/292) of patients.

Neither nuclear pRB1, cytoplasmic pRB1, nor their co-expression was associated with the clinicopathological variables (Supplementary Tables S19–S21). Nuclear pRB1 was not significantly associated with PFS (*p* = 0.127) (Figure 3E) but was significantly linked with OS (*p* = 0.01) (Figure 3F). Cytoplasmic pRB1 did not influence survival (Supplementary Figure S8A,C). High nuclear/high cytoplasmic pRB1 expression was significantly linked with PFS (*p* = 0.046) (Supplementary Figure S8B) and OS (*p*= 0.017) (Supplementary Figure S8D). 

### 2.8. Multivariate Analysis

The multivariate analysis included tumour stage and CDK4, CDK6, CDK2, cyclin D1, cyclin E1, RB1 and pRB1 protein expression. As shown in Table 2, cyclin E1 and tumour stage were independently associated with PFS. Meanwhile, tumour stage only was independently associated with OS (Table 2).

### 2.9. Cyclin E1 and CDK2 Protein Co-Expression

**Figure 4:**
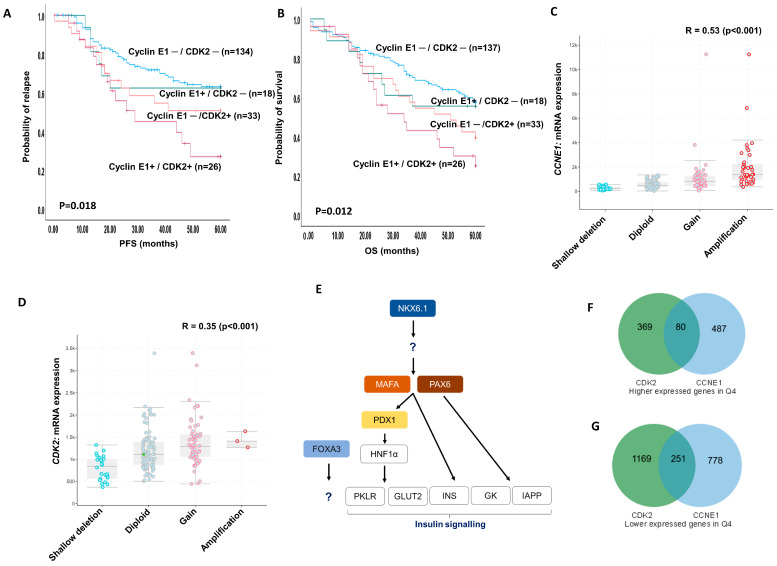
**Cyclin E1–CDK2 co-expression and bioinformatics**. (**A**) Kaplan–Meier curve for cyclin E1 and CDK2 co-expression and progression-free survival. (**B**) Kaplan–Meier curve for cyclin E1 and CDK2 co-expression and overall survival. (**C**,**D**) cBioPortal GISTIC analysis for CCNE1 and CDK2 shows positive correlation between copy number alterations and mRNA levels in the Ovarian Serous Cystadenocarcinoma TCGA cohort. (**E**) Insulin signalling pathway showing higher mRNA levels of transcription factors (shaded, coloured boxes) associated with more strongly CCNE1-expressing tumours (edited from Maturity onset diabetes of the young KEGG pathway hsa04950). (**F**) Differential gene expression comparison between CCNE1 and CDK2 genes with a higher expression in quartile 4 tumours. (**G**) Differential gene expression comparison between CCNE1 and CDK2 genes with a lower expression in quartile 4 tumours.

As shown in Table 3, we observed a positive correlation between CDK2 protein expression and cyclin E1 protein expression in our clinical cohort. High nuclear cyclin E/high nuclear CDK2 was significantly associated with poor PFS (*p* = 0.018) (Figure 4A) and a shorter OS (*p* = 0.012) (Figure 4B).

### 2.10. Bioinformatics in Ovarian TCGA

As cyclin E1 was independently associated with poor PFS, we proceeded to conduct bioinformatics analysis on the Ovarian Serous Cystadenocarcinoma TCGA cohort. Utilising cBioPortal, we observed that 22% of ovarian tumours (TCGA, Firehose Legacy, *n* = 311) had *CCNE1* gene amplification (*n* = 67/311). As cyclin E1 is involved in the activation of CDK2, we also evaluated CDK2 status. For *CDK2*, 1% of tumours harboured genetic alterations (9 amplification, 1 missense mutation). Using GISTIC analysis on 182 patient samples that had mRNA expression data, we found that the copy number variation showed a positive correlation with the mRNA levels for both *CCNE1* and *CDK2* (Pearson’s correlations of 0.53 (*p* < 0.001) and 0.35 (*p* < 0.001), respectively (Figure 4C,D)). Differential gene expression analysis was performed utilising TCGA-OV RNA-Seq data for 379 samples to analyse genes expressed lower or higher in the tumours with low or high levels of *CCNE1* or *CDK2* (Q1 vs. Q4). At the mRNA level, there was a weak significant positive correlation between *CCNE1* and *CDK2* (Pearson’s correlation of 0.208, *p* = 0.00004). For tumours with high *CCNE1*, 567 genes were expressed more highly and 1029 genes had a lower expression than in the low-*CCNE1* tumours, with CCNE1 differentially expressed with a log2 fold change of 3.01 (FDR-*p* <0.05) (Supplementary Data S1). Pathway analysis was performed for the higher and lower genes separately. Two significant pathways, hsa04080, Neuroactive ligand-receptor interaction, and hsa04950, Maturity onset diabetes of the young (MODoY; with transcription factors *FOXA3*, *NKX6-1*, *MAFA*, *PAX6*, *PDX1*), were identified for the genes expressed more highly in the high-*CCNE1* tumours. Of interest are the transcription factors identified in the MODoY pathway, as these genes lead to insulin signalling and release (Figure 4E). The relevance of high CCNE1 and the use of metformin has recently been shown, with metformin treatment reducing CCNE1 levels [20]. For the genes with a lower expression in the Q4 tumours, the relevant pathways were hsa05218, melanoma; hsa04014, Ras signalling pathway; hsa05226, Gastric cancer, with *CCND1* and *FGF*s highlighted in these pathways.

In the *CDK2* analysis, 449 genes had a higher expression and 1420 genes were expressed less in the tumours with high *CDK2. CDK2* was differentially expressed with a log2 fold change of 1.1 (Supplementary Data S2). No pathways were identified for the genes expressed more highly in the Q4 tumours, and the genes with a lower expression were linked to the pathways hsa03010 ribosome (RPL genes) and hsa05034 alcoholism (Histone cluster genes). A comparison of differentially expressed genes was then carried out for *CCNE1* and *CDK2*, where a subset of genes was identified to be altered in the high-expression tumours (Figure 4F,G). One of the differentially expressed genes expressed at a higher level in the CCNE1/CDK2-high tumours was *FOXA1* (FDR-*p* value < 0.05; log2 fold changes of 2.2 and 1.1, respectively). The pioneer transcription factor FOXA1 has been shown to be involved in ovarian tumour progression [21,22]. 

## 3. Discussion

Ovarian cancers are characterised by cell cycle dysregulation, replication stress and genomic instability. The cyclin D1/E1–cyclin-dependent kinase (CDK2/4/6)–retinoblastoma (RB1/pRB1) pathway is critical to the regulation of G1-S cell cycle progression. In the nucleus, CDK2, CDK4 and CDK6 are required for G1/S cell cycle progression [23]. However, emerging evidence indicates that CDK4 and CDK6 also have cytoplasmic functions that influence pro-metastatic features, including migration, invasion, angiogenesis and differentiation [23,24,25,26,27,28]. CDK4 and CDK6 may also have roles during protein ubiquitination and thereby control protein stability. CDK4 and CDK6 may also regulate gene transcription, senescence and cell metabolism. Transforming growth factor-β can induce CDK2 re-localisation to the cytoplasm, which is associated with the dephosphorylation of RB1 [29]. CDK2 hyper-phosphorylates RB1 and inactivates it. CDK2 is also involved in the phosphorylation of several transcription factors, including SMAD3, FOXM1, FOXO1 and MYC. Besides a role in cell cycle regulation, CDK2 is also involved during DNA replication, adaptive immune response, cell differentiation and apoptosis [30]. In the current study, we have comprehensively investigated the sub-cellular localisation and clinicopathological significance of cyclins D1/E1, CDK2/4/6 and RB1/pRB1 in a cohort of clinical ovarian cancers. Nuclear CDK2 and nuclear cyclin E1 overexpression were linked with poor PFS and poor OS. Our data concur with a previous study which showed that high expression levels of CDK2 and cyclin E1 were independently related to a poor prognosis [31]. Nuclear CDK6 was associated with poor OS. Interestingly, cytoplasmic expression of CDK4, cyclin D1 and cyclin E1 also has predictive and/or prognostic significance in ovarian cancers. However, in a previous study of 103 ovarian tumours, no significant association was observed between CDK4 expression and clinicopathological parameters [32]. Previous evidence showed that high expression of RB1 is associated with a poor prognosis in advanced-stage ovarian carcinoma patients [33]. Similarly, in our study, we found a correlation between pRB1 and poor survival. In line with our study, Kommoss et al. also found that high protein expression levels of pRB1 were associated with an incremental deterioration in prognosis [33]. In the multivariate analysis, we also showed that nuclear cyclin E1 was an independent predictor of poor PFS. Tumours with high nuclear cyclin E1/high nuclear CDK2 also had a worse PFS and OS. The detailed bioinformatics in the TCGA cohort showed a positive correlation between cyclin E1 and CDK2. We have also shown the CCNE1-overexpressing tumours are enriched in genes involved in insulin signalling and release. However, a limitation to our study is that the immunohistochemical and transcriptomic analyses were conducted on two independent cohorts. Nevertheless, taken together, our data not only show alterations in sub-cellular localisation but also demonstrate the predictive and/or prognostic significance of the cyclin D1/E1–CDK2/4/6–RB1 pathway in ovarian cancer.

In the current study, low nuclear cyclin D1 showed a significant association with serous carcinoma and a high tumour grade but did not influence survival. However, low cytoplasmic cyclin D1 was linked with poor OS. In a previous study of 81 ovarian tumours [34], cyclin D1 overexpression was observed in 89% of cases [nucleus and cytoplasm co-expression in 30% and localisation exclusively in the cytoplasm in 59%]. However, no association with the clinicopathological features or survival outcomes was observed in that study [34]. In another immunohistochemical study of 134 ovarian tumours, cyclin D1 overexpression was associated with poor survival outcomes [35]. In a further study of 50 ovarian tumours, cyclin D1 overexpression was observed in 70% of tumours and linked with poor survival [36]. Additional studies have observed that high nuclear expression of cyclin D1 has prognostic significance in advanced ovarian cancer [37,38].

In the current study, we have shown that cytoplasmic CDK4 was associated with an aggressive phenotype and poorer survival. However, in a previous study of 103 ovarian tumours, no significant correlation was observed between CDK4 expression and the clinicopathological parameters [32]. Genomic alterations in CDK4 and CDK6 have been reported in ovarian cancer [39]. A bioinformatics study investigated alterations in the CDK4/6 pathway in an ovarian TCGA cohort [39]. CDKN2A (p16^INK4a^), which is known to inhibit CDK4 and CDK6, was deleted or downregulated in 21% of the ovarian tumours. Amplification of or mRNA overexpression of CDK4, CDK6 and/or cyclin D1 was observed in 16% of tumours. RB1 was deleted or downregulation was seen in 17% of tumours. Pre-clinically, ribociclib therapy had anti-cancer activity in both platinum-sensitive and platinum-resistant ovarian cancer cell lines [39]. 

Here, we show high nuclear CDK6 was associated with poor OS. In a pre-clinical study [12], gene silencing of or the pharmacological inhibition of CDK6 increased the platinum sensitivity in ovarian cancer cell lines. CDK6 phosphorylation following platinum treatment stabilised the FOXO3 transcription factor, thereby inducing ATR transcription. Clinically, high CDK6 and FOXO3 expression was associated with poor survival in that study [12]. Similarly, another study also showed that increased CDK6 protein expression was associated with a poor prognosis [40].

Our observation that cyclin E1 (CCNE1) overexpression is associated with poor survival outcomes concurs with previous studies. In a study of 103 cases, cyclin E overexpression was associated with poor survival [41]. In another study, *CCNE1* amplification was observed in 20.4% of cases. There was a positive correlation between the *CCNE1* copy number and CCNE1 (cyclin E1) protein expression. Although CCNE1 gene amplification was associated with poor survival, cyclin E1 protein overexpression was not in that study [42]. Surprisingly, in another study, high CCNE1 expression (seen in 25% of tumours) was linked with better survival [43]. In a clear cell ovarian cancer study, cyclin E1 overexpression was observed in 23.3% of tumours and associated with poor survival outcomes [44]. Cyclin E1 overexpression was also associated with platinum resistance in another immunohistochemical study in 110 ovarian cancers [45]. 

In the current study, we have shown that tumours with cyclin E1 and CDK2 overexpression have a worse PFS and OS. The data suggest that cyclin-E1-overexpressing tumours would be suitable to target using a CDK2 inhibitor. A recent pre-clinical study provides evidence that such as approach is feasible. The investigators observed that cyclin E1 (CCNE1) was overexpressed in 30% of established ovarian cancer cell lines, and such cancer cells were 40 times more sensitive to SNS-032 therapy, a CDK2 small molecule inhibitor [46]. Importantly, early-phase clinical trials of CDK2 inhibitors such as SNS-032, fadraciclib, BLU-222 and dinaciclib are currently underway in solid tumours [47]. Whether such an approach will have a positive clinical impact on ovarian cancer remains to be established.

## 4. Conclusions

Our data taken as a whole provide clear evidence that cyclin E1 and CDK2 overexpression is linked to a worse PFS and OS. CDK2 targeting in cyclin-E1-overexpressing ovarian cancer will have a positive clinical impact.

## 5. Materials and Methods

### 5.1. Study Cohort

The expression of CDK4, CDK6, CDK2, cyclin D1, cyclin E1 and pRB1 was evaluated on tissue microarrays of 300 consecutive epithelial ovarian cancers treated at Nottingham University Hospitals (NUH) between 1997 and 2010. The patients were comprehensively staged as per the International Federation of Obstetricians and Gynaecologists (FIGO) Staging System for Ovarian Cancer. Overall survival was calculated from the operation date until the time of death or the last date of follow-up, at which point any remaining survivors were censored. All the patients received platinum-based chemotherapy. Platinum resistance was defined as patients who had progression during first-line platinum chemotherapy or a relapse within 6 months after the completion of chemotherapy. Progression-free survival was calculated from the date of the initial surgery to disease progression or from the date of the initial surgery to the last date known to be progression-free for those censored. The patient demographics are summarised in Supplementary Table S1. 

The Tumour Marker Prognostic Studies (REMARK) criteria, recommended by McShane et al. [48], were followed throughout this study. This study was carried out in accordance with the Declaration of Helsinki, and ethical approval was obtained from the Nottingham Research Ethics Committee (REC Approval Number 06/Q240/153).

### 5.2. Antibodies and Western Blot 

Prior to immunohistochemistry (IHC) staining of the tissue sections, the specificity of the Anti-CDK4 antibody [EPR4513-32-7] ab108357 (abcam, Cambridge, UK), Anti-Cdk6 antibody [EPR4515] ab12482 (abcam, Cambridge, UK), Anti-Cdk2 antibody [E304] ab32147 (abcam, Cambridge, UK), anti-Cyclin D1 antibody [SP4] ab16663 (abcam, Cambridge, UK) and Anti-Rb1/Retinoblastoma protein phospho (Ser795) and Anti-Rb1/Retinoblastoma protein (ARG51103) antibodies (Cambridge Biosciences, Cambridge, UK) was validated via Western blotting using cell lysates of the POE1, POE4, A2780 and A2780cis ovarian cancer cell lines, obtained from the American Type Culture Collection, Rockville, MD, USA. The Anti-Cyclin E1 antibody [EP435E] ab33911 was validated in the COV318, COV362 and OVCAR3 ovarian cancer cell lines, obtained from the American Type Culture Collection, Rockville, MD, USA. The extracts were quantified using a BCA protein quantification kit, and the protein levels were checked using Western blot. The samples were run on SDS Bolt bis-tris gel (4–12%). The membranes were then incubated with the primary antibodies as follows: The CDK4 and CDK6 primary antibodies were used at 1:750 dilution; CDK2, Cyclin D1, Cyclin E1, pRB1 and RB1 were used at 1.1000 dilution; ß-actin, 1:1000, ab8226; GADPH, 1:1000, ab9485. The membranes were incubated overnight with the primary antibodies. The membranes were then washed and incubated with infrared-dye-labelled secondary antibodies (LI-COR, Cambridge, UK) (IRDye 800CW Donkey Anti-Rabbit IgG (926-32213) (LI-COR, Cambridge, UK) and IRDye 680CW Donkey Anti-Mouse IgG (926–68,072) (LI-COR, Cambridge, UK) at a dilution of 1:10,000 for 60 min. The membranes were scanned using a LI-COR Odyssey machine (700 and 800 nm, Cambridge, UK) to determine the protein levels. Supplementary Figure S1 shows the specificity of each marker using Western blot.

### 5.3. Tissue Microarrays (TMAs) and Immunohistochemistry (IHC)

The tumour samples were arrayed into tissue microarrays (TMAs), constructed with 2 replicate 0.6 mm cores from the tumours. Immunohistochemical staining was performed using the Thermo Fisher Scientific Shandon Sequenza chamber system (REF: 72110017, Cheshire, UK), in combination with the Novolink Max Polymer Detection System (RE7280-K: 1250 tests, Buffalo Grove, IL, USA) and the Leica Bond Primary Antibody Diluent (AR9352, Buffalo Grove, IL, USA), each used according to the manufacturer’s instructions (Leica Microsystems, Buffalo Grove, IL, USA). The TMA slides were deparaffinised with xylene and then rehydrated using five decreasing concentrations of alcohol (100%, 90%, 70%, 50% and 30%) for two minutes each. Pre-treatment, antigen retrieval was carried out on the TMA sections using sodium citrate buffer (pH of 6.0), which were heated at 95 C in a microwave (Whirlpool JT 359 Jet Chef, 1000 W, UK) for 20 min. A set of slides was incubated with the primary antibodies: CDK4 and CDK6 at a dilution of 1:100 for 60 min at room temperature, CDK2 at a dilution of 1.100 for 90 min, cyclin D1 at a dilution of 1.25 for 60 min, cyclin E1 at a dilution of 1.300 for 45 min and both pRB1 and RB1 at a dilution of 1.500 for 60 min. A negative control and positive controls were included in each run. Diaminobenzidine (DAB) was used to visualise the immunochemical staining, and finally, counterstaining with Meyer’s Haematoxylin was performed.

### 5.4. Evaluation of the Immune Staining

The cores of the TMAs were assessed in terms of their suitability for scoring. For example, cores with less than 20% tumour were excluded from the study. For each sample, visual assessment of the staining was performed, and the subcellular localisation of each marker was identified (nuclear, cytoplasm, cell membrane or mixed). The intensities of subcellular localisation were evaluated for each marker as follows: 0 = no staining, 1 = weak staining, 2 = moderate staining, 3 = strong staining. The percentage of protein expression was evaluated (0–100%). In addition, the histochemical score (H-score) (range of 0–300) was calculated by multiplying the intensity of the staining and the percentage of the staining. CDK4, CDK6, CDK2, cyclin D1, cyclin E1, RB1 and pRB1 showed nuclear and cytoplasmic subcellular localisation. Therefore, the H-score was evaluated for the nuclear and cytoplasmic expression of each marker. Not all the cores within the TMAs were included in the IHC analysis due to missing cores or the absence of tumour cells. X-tile bioinformatics software version 3.6.1 (School of Medicine, Yale University, New Haven, CT, USA) was used to generate the best cut-offs for both the nuclear and cytoplasmic expression of each marker based on the patient outcomes [49]. This software randomly divides the patient cohort into two separate equal sets, training and validation sets, by producing separate lists of “censored” and “uncensored” observations, ranked by the patients’ follow-up time [49]. The optimal cut-offs were determined by locating the brightest pixel on the X-tile plot diagram of the training set [49]. The statistical significance was tested by applying the obtained cut-off to the validation set.

### 5.5. CDK4, CDK6, CDK2, Cyclin D1, Cyclin E1 and RB1 Transcripts in Ovarian Cancers

The differential expression of the normal versus ovarian cancer tissue transcripts was evaluated using TNMplot.com [50]. The predictive and prognostic significance of the mRNA expression of CDK4, CDK6, CDK2, cyclin D1, cyclin E1 and RB1 was assessed in a publicly available online gene expression dataset of 1259 ovarian cancer patients treated with platinum-based chemotherapy from 15 previously published studies and available at www.kmplot.com [51]. 

### 5.6. Statistical Analysis

Statistical Package for the Social Sciences software v.27.0 (SPSS, Chicago, IL, USA) was used for the statistical analysis. Correlations with the clinical and pathological characteristics using categorised data were calculated using the Chi-square test. Bonferroni correction was applied to multiple comparisons. To define the best cut-off points for the studied markers, we used X-tile software. Based on X-tile, the best cut-offs for the nuclear and cytoplasmic expression of CDK4 were 0 and 80, while for nuclear and cytoplasmic CDK6, they were 32 and 4, respectively. For CDK2, the best cut-offs for nuclear and cytoplasmic expression were 5 and 26, accordingly. The optimal cut-offs for the nuclear and cytoplasmic expression of cyclin D1 were 180 and 10, respectively, while they were 110 and 80 for the nuclear and cytoplasmic expression of Cyclin E1, accordingly. The best cut-offs for the nuclear and cytoplasmic expression of RB1 were 10 and 150, respectively. In addition, the best cut-offs for the nuclear and cytoplasmic expression of pRB1 were 200 and 110, respectively. All the tests were 2-tailed. The survival rates were determined using the Kaplan–Meier method and compared using the log-rank test. A *p* value < 0.05 was identified as statistically significant.

### 5.7. Bioinformatics

cBioPortal was used to assess the *CCNE1* and *CDK2* mutations and copy number variations in the Ovarian Serous Cystadenocarcinoma TCGA cohort [TCGA, Firehose Legacy, 311 samples/patients; [52]]. The TCGA ovarian cancer (TCGA, Nature 2011) RNA-Seq expression data for 379 OV specimens were obtained from GDC (https://portal.gdc.cancer.gov/, accessed on 06 November 2023). The tumour specimens were ranked from the lowest to the highest expression for *CCNE1/CDK2* and placed into quartiles. *CCNE1* and *CDK2* were analysed separately. Quartile 1 (Q1) contained low-*CCNE1*/*CDK2* tumours, while quartile 4 (Q4) contained high-*CCNE1*/*CDK2* tumours. Differential analysis between *CCNE1/CDK2* Q1 and Q4 was performed using DESeq2, which normalised the dataset and calculated the differential gene expression profile between Q1 and Q4 [53]. Differentially expressed genes (DEGs) were significant when the log2 FC ≥ 1 and the FDR-corrected *p*-value < 0.05. Pearson’s correlation coefficient was calculated for the RNA-Seq counts for the OV-TCGA dataset between *CCNE1* and *CDK2*, with a significance of *p* < 0.05. To assess the significance of the DEGs, over-representation pathway analysis of the genes with a lower or higher expression in Q1 vs. Q4 was performed using WebGestalt v. 2019 [54]. Significant pathways were FDR-corrected as <0.05.

## Figures and Tables

**Table 1:** Nuclear CDK2 expression and clinicopathological parameters.

Parameters	Nuclear CDK2 Expression			
	Low (H Score ≤ 5)	High (H Score > 5)	*p* Value	Adjusted *p* Value
	N (%)	N (%)		
**Pathology Type**				
Serous	108 (51%)	53 (65%)	**0.026**	0.06
Mucinous	38 (18%)	5 (6%)		
Endometriod	33 (15%)	7 (9%)		
Clear Cell	14 (6%)	10 (12%)		
Other	9 (4%)	2 (3%)		
Mixed	12 (6%)	4 (5%)		
**Pathology Grade**				
Low	31 (17%)	7 (10%)	**0.001**	**0.005**
Med	47 (26%)	7 (10%)		
High	101 (57%)	58 (80%)		
**Pathology Stage**				
1	89 (44%)	23 (28%)	**0.031**	0.09
2	32 (16%)	11 (14%)		
3	74 (37%)	45 (56%)		
4	7 (3%)	2 (2%)		
**Residual Tumour** **Post S** **urgery**				
Non	142 (73%)	48 (65%)	0.511	0.511
<1 cm	22 (11%)	11 (15%)		
1–2 cm	7 (4%)	5 (7%)		
>2 cm	24 (12%)	10 (13%)		
**Platinum** **Sensitivity**			0.312	0.31
Sensitive	168 (92%)	60 (88%)		
Resistant	14 (8%)	8 (12%)		

Significant *p* values are in bold. Adjusted *p* value was calculated using Bonferroni correction.

**Table 2:** Multivariate analysis of CDK4, CDK6, CDK2, Cyclin D1, Cyclin E1, RB1 and pRB1.

Parameters	Progression Free Survival			Overall Survival		
	Hazard Ratio	95% (CI)	*p*-Value	Hazard Ratio	95% (CI)	*p*-Value
CDK4	0.63	0.29–1.3	0.23	0.78	0.41–1.5	0.46
CDK6	0.81	0.32–2.1	0.65	1.4	0.68–2.8	0.38
CDK2	0.82	0.35–1.9	0.64	0.99	0.45–2.1	0.98
Cyclin D1	0.64	0.07–5.3	0.68	0.77	0.16–3.5	0.73
Cyclin E1	2.11	1.1–4.2	**0.03**	1.4	0.73–2.8	0.32
pRB1	1.69	0.68–4.2	0.26	1.7	0.73–4.5	0.19
RB1	1.33	0.71–2.5	0.36	0.89	0.5–1.5	0.67
Pathology stage	1.9	1.3–2.8	**<0.0001**	2.4	1.69–3.41	**<0.0001**

95% CI, 95% confidence interval. Significant *p* values are in bold.

**Table 3:** Relationship between CDK2 and cyclin E1.

Variables	Cyclin E1 Expression		*p*-Value
	Low	High	
**CDK2 expression**			
**Low**	159 (81%)	38 (19%)	34.7
**High**	20 (39%)	31 (61%)	**<0.0001**

## Data Availability

The data supporting the study can be found in the Supplementary Materials File, and the corresponding author can make any materials available upon request. The aggregate data from the referenced datasets are available from the corresponding author on reasonable request.

## Supplementary Materials

The following supporting information can be downloaded at https://www.mdpi.com/article/10.3390/ijms25074060/s1.
