# Supplementary material for: The Clinicopathological Significance of the Cyclin D1/E1–Cyclin-Dependent Kinase (CDK2/4/6)–Retinoblastoma (RB1/pRB1) Pathway in Epithelial Ovarian Cancers
Source: Int J Mol Sci. 2024 Apr 5;25(7):4060. doi: 10.3390/ijms25074060 (PMC11012085; doi:10.3390/ijms25074060)
Supplement: Supplementary file 1 [file ijms-25-04060-s001.zip › Supplementary materials (2)_Revision 1_sm.pdf]

Supplementary Table S1. Clinicopathological characteristics of the ovarian cancer cohort.

Parameters	Number (%)
**Surgical Pathology Type**	
**Serous**	154 (56%)
**Mucinous**	39 (14%)
**Endometriod**	42 (15%)
**Clear Cell**	16 (6%)
**Other**	13 (5%)
**Mixed**	12 (4%)
**Surgical Pathology Grade**	
**Low**	40 (17%)
**Med**	57 (25%)
**High**	134 (58%)
**Surgical Pathology Stage**	
**1**	101 (39%)
**2**	43 (17%)
**3**	108 (41%)
**4**	10 (3%)
**Residual Tumour Following Surgery**	
**Non**	170 (69%)
**<1cm**	27 (11%)
**1-2cm**	13 (5%)
**>2cm**	38 (15%)
**Platinum Sensitivity**	
**Sensitive**	217 (92%)
**Resistant**	20 (8%)

Supplementary Table S2: Cytoplasmic CDK2 expression and clinicopathological parameters.

Parameters	Cytoplasmic CDK2 expression		*p value*	*Adjusted p value*
	Low	*High*		
	N (%)	*N (%)*		
**Pathology Type**				
Serous	103 (48%)	58 (72%)	**0.001**	**0.005**
Mucinous	40 (19%)	3 (4%)		
Endometriod	31 (15%)	9 (11%)		
Clear Cell	22 (10%)	2 (2%)		
Other	7 (3%)	4 (5%)		
Mixed	11 (5%)	5 (6%)		
**Pathology Grade**				
Low	32 (18%)	6 (8%)	0.069	0.069
Med	39 (22%)	15 (19%)		
High	104 (60%)	55 (73%)		
**Pathology Stage**				
1	92 (45%)	20 (25%)	**0.0271**	0.11
2	30 (14%)	13 (16%)		
3	77 (38%)	42 (54%)		
4	6 (3%)	3 (5%)		
**Residual tumour post Surgery**				
Non	149 (75%)	41 (58%)	**0.036**	0.07
<1cm	20 (10%)	13 (18%)		
1-2cm	6 (3%)	6 (8%)		
>2cm	23 (12%)	11 (16%)		
**Platinum Sensitivity**			**0.027**	0.08
**Sensitive**	164 (89%)	64 (97%)		
**Resistant**	20 (11%)	2 (3%)		

Significant p values are in bold.

Adjusted p value was calculated using Bonferroni correction.

Supplementary Table S3: Nuclear and cytoplasmic CDK2 co-expression and clinicopathological parameters.

**Parameters**	**Nuclear (n) and cytoplasmic (c) co-expression**				*p value*	*Adjusted p value*
	Low (n)-low (c)	*High (n)-low (c)*	*High (n)-high (c)*	*Low (n)-high (c)*		
	N (%)	*N (%)*	*N (%)*	*N (%)*		
**Pathology Type**						
Serous	73 (45%)	36 (67%)	23 (83%)	29 (56%)	**0.001**	**0.007**
Mucinous	36 (22%)	2 (4%)	1 (3%)	4 (8%)		
Endometriod	26 (16%)	7 (13%)	2 (8%)	5 (9%)		
Clear Cell	12 (8%)	2 (4%)	0 (0%)	10 (19%)		
Other	6 (4%)	3 (5%)	1 (3%)	1 (2%)		
Mixed	8 (5%)	4 (7%)	1 (3%)	3 (6%)		
**Pathology Grade**						
Low	26 (20%)	5 (10%)	1 (4%)	6 (13%)	**0.004**	**0.02**
Med	36 (28%)	11 (21%)	4 (15%)	3 (7%)		
High	67 (52%)	35 (69%)	21 (81%)	36 (80%)		
**Pathology Stage**						
1	75 (49%)	14 (27%)	6 (21%)	17 (33%)	**0.022**	0.08
2	24 (16%)	8 (16%)	5 (18%)	6 (11%)		
3	49 (32%)	26 (51%)	17 (61%)	27 (52%)		
4	4 (3%)	3 (6%)	0 (0%)	2 (4%)		
**Residual tumour post-surgery**						
None	115 (77%)	27 (58%)	14 (56%)	34 (71%)	0.081	0.24
<1cm	15 (10%)	7 (15%)	6 (24%)	5 (10%)		
1-2cm	4 (3%)	3 (6%)	3 (12%)	2 (4%)		
>2cm	15 (10%)	10 (21%)	2 (8%)	7 (15%)		

**Platinum Sensitivity**					0.176	0.34
Sensitive	126 (91%)	42 (93%)	22 (100%)	38 (84%)		
Resistant	12 (9%)	3 (7%)	0 (0.0%)	7 (16%)		

Significant p values are in bold.

Adjusted p value was calculated using Bonferroni correction.

Supplementary Table S4: Nuclear CDK4 expression and clinicopathological parameters.

Parameters	Nuclear CDK4 expression		*p value*	*Adjusted p value*
	Low	*High*		
	N (%)	*N (%)*		
**Surgical Pathology Type**				
**Serous**	137 (57%)	26 (54%)	0.894	0.894
**Mucinous**	35 (15%)	5 (11%)		
**Endometriod**	32 (13%)	7 (15%)		
**Clear Cell**	18 (7%)	4 (8%)		
**Other**	8 (3%)	3 (6%)		
**Mixed**	12 (5%)	3 (6%)		
**Surgical Pathology Grade**				
**Low**	34 (16%)	7 (16%)	0.492	0.492
**Med**	41 (20%)	12 (28%)		
**High**	131 (64%)	24 (56%)		
**Surgical Pathology Stage**				
**1**	97 (42%)	16 (34%)	0.6	0.6
**2**	36 (15%)	10 (21%)		
**3**	91 (39%)	20 (43%)		
**4**	9 (4%)	1 (2%)		
**Residual Tumour Following Surgery**				
**Non**	155 (71%)	30 (68%)	0.687	0.687
**<1cm**	25 (11%)	6 (14%)		
**1-2cm**	13 (6%)	1 (2%)		
**>2cm**	27 (12%)	7 (16%)		
**Platinum Sensitivity**			0.856	0.856
**Sensitive**	190 (91%)	36 (90%)		
**Resistant**	19 (10%)	4 (10%)		

Significant p values are in bold.

Adjusted p value was calculated using Bonferroni correction.

Supplementary Table S5: Cytoplasmic CDK4 expression and clinicopathological parameters.

**Parameters**	**Cytoplasmic CDK4 expression**		***p value***	***Adjusted P value***
	Low	***High***		
	N (%)	***N (%)***		
**Pathology Type**				
Serous	91 (46%)	72 (79%)	**<0.0001**	**<0.0001**
Mucinous	38 (19%)	2 (2%)		
Endometriod	30 (15%)	9 (10%)		
Clear Cell	20 (10%)	2 (2%)		
Other	8 (4%)	3 (3%)		
Mixed	11 (6%)	4 (4%)		
**Pathology Grade**				
Low	38 (23%)	3 (4%)	**<0.0001**	**<0.0001**
Med	41 (24%)	12 (14%)		
High	88 (53%)	67 (82%)		
**Pathology Stage**				
1	89 (47%)	24 (27%)	**0.001**	**0.003**
2	32 (17%)	14 (15%)		
3	60 (32%)	51 (56%)		
4	8 (4%)	2 (2%)		
**Residual tumour post-surgery**				
None	140 (76%)	45 (57%)	**0.018**	**0.036**
<1cm	17 (9%)	14 (18%)		
1-2cm	7 (4%)	7 (9%)		
>2cm	21 (11%)	13 (16%)		
**Platinum Sensitivity**			0.641	0.641

Sensitive	158 (91%)	68 (90%)
Resistant	15 (9%)	8 (10%)

Significant p values are in bold.

Adjusted p value was calculated using Bonferroni correction.

Supplementary Table S6: Nuclear and cytoplasmic CDK4 co-expression and clinicopathological parameters

Parameters	CDK4 Nuclear (n) and cytoplasmic (c) co-expression				*p value*	*Adjusted P value*
	**Low (n)-low (c)**	**High (n)-low (c)**	**High (n)-high (c)**	**Low (n)-high (c)**		
	N (%)	***N (%)***	***N (%)***	***N (%)***		
**Pathology Type**						
Serous	77 (47%)	14 (42%)	12 (79%)	60 (78%)	**0.003**	**0.01**
Mucinous	33 (20%)	5 (15%)	0 (0%)	2 (2%)		
Endometriod	24 (14%)	6 (18%)	1 (7%)	8 (10%)		
Clear Cell	16 (10%)	4 (12%)	0 (0%)	2 (3%)		
Other	6 (4%)	2 (6%)	1 (7%)	2 (3%)		
Mixed	9 (5%)	2 (6%)	1 (7%)	3 (4%)		
**Pathology Grade**						
Low	31 (23%)	7 (23%)	0 (0%)	3 (4%)	**0.001**	**0.007**
Med	32 (23%)	9 (30%)	3 (23%)	9 (13%)		
High	74 (54%)	14 (47%)	10 (77%)	57 (83%)		
**Pathology Stage**						
1	75 (48%)	14 (44%)	2 (13%)	22 (29%)	**0.01**	0.05
2	24 (15%)	8 (25%)	2 (13%)	12 (16%)		
3	51 (33%)	9 (28%)	11 (74%)	40 (52%)		
4	7 (4%)	1 (3%)	0(0%)	2 (3%)		
**Residual Tumour post-surgery**						
None	114 (74%)	26 (84%)	4 (31%)	41 (62%)	**0.03**	0.12
<1cm	15 (10%)	2 (6%)	4 (31%)	10 (15%)		
1-2cm	7 (4%)	0 (0%)	1 (7%)	6 (9%)		

**>2cm**	18 (12%)	3 (10%)	4 (31%)	9 (14%)		
**Platinum Sensitivity**					0.869	0.86
Sensitive	133 (91%)	25 (93%)	11 (85%)	57 (90%)		
Resistant	13 (9%)	2 (7%)	2 (15%)	6 (10%)		

Significant p values are in bold.

Adjusted p value was calculated using Bonferroni correction.

Supplementary Table S7: Nuclear CDK6 expression and clinicopathological parameters

**Parameters**	**Nuclear CDK6 expression**		*p value*	*Adjusted p value*
	Low	*High*		
	N (%)	*N (%)*		
**Surgical Pathology Type**				
Serous	108 (53%)	46 (65%)	**0.04**	0.2
Mucinous	33 (16%)	6 (8%)		
Endometriod	35 (17%)	7 (10%)		
Clear Cell	13 (6%)	3 (4%)		
Other	6 (3%)	7 (10%)		
Mixed	10 (5%)	2 (3%)		
**Surgical Pathology Grade**				
Low	25 (15%)	15 (23%)	0.306	0.306
Med	43 (26%)	14 (22%)		
High	99 (59%)	35 (55%)		
**Surgical Pathology Stage**				
1	79 (40%)	22 (33%)	0.08	0.4
2	37 (19%)	6 (9%)		
3	73 (37%)	35 (53%)		
4	7 (4%)	3 (5%)		
**Residual Tumour post-surgery**				
None	132 (72%)	38 (60%)	0.161	0.32
<1cm	21 (11%)	6 (10%)		
1-2cm	9 (5%)	4 (6%)		
>2cm	23 (12%)	15 (24%)		
**Platinum Sensitivity**				

Sensitive	166 (93%)	51 (86%)	0.10	0.4
Resistant	12 (7%)	8 (14%)		

Significant p values are in bold.

Adjusted p value was calculated using Bonferroni correction.

Supplementary Table S8: Cytoplasmic CDK6 expression and clinicopathological parameters.

**Parameters**	**Cytoplasmic CDK6 expression**		*p value*	*Adjusted P value*
	Low	*High*		
	N (%)	*N (%)*		
**Surgical Pathology Type**				
**Serous**	140 (55%)	14 (61%)	0.765	0.765
**Mucinous**	36 (14%)	3 (13%)		
**Endometriod**	39 (16%)	3 (13%)		
**Clear Cell**	15 (6%)	1 (4%)		
**Other**	13 (5%)	0 (0%)		
**Mixed**	10 (4%)	2 (9%)		
**Surgical Pathology Grade**				
**Low**	38 (18%)	2 (10%)	0.402	0.402
**Med**	53 (25%)	4 (19%)		
**High**	119 (57%)	15 (71%)		
**Surgical Pathology Stage**				
**1**	98 (41%)	3 (13%)	**0.02**	0.4
**2**	40 (17%)	3 (13%)		
**3**	92 (38%)	16 (70%)		
**4**	9 (4%)	1 (4%)		
**Residual Tumour Following Surgery**				
**Non**	160 (70%)	10 (50%)	0.261	0.261
**<1cm**	23 (10%)	4 (20%)		
**1-2cm**	11 (5%)	2 (10%)		
**>2cm**	34 (15%)	4 (20%)		
**Platinum Sensitivity**			0.85	0.85
**Sensitive**	198 (92%)	19 (90%)		
**Resistant**	18 (8%)	2 (10%)		

Significant p values are in bold.

Adjusted p value was calculated using Bonferroni correction.

Supplementary Table S9: Nuclear and cytoplasmic CDK6 co-expression and clinicopathological parameters.

Parameters	CDK6 Nuclear (n) and cytoplasmic (c) co-expression				*p value*	*Adjusted P value*
	**Low (n)-low (c)**	**High (n)-low (c)**	**High (n)-high (c)**	**Low (n)-high (c)**		
	N (%)	*N (%)*	*N (%)*	*N (%)*		
**Pathology Type**						
Serous	97 (52%)	43 (64%)	3 (75%)	11 (58%)	0.360	0.360
Mucinous	30 (16%)	6 (9%)	0 (0%)	3 (16%)		
Endometriod	33 (18%)	6 (9%)	1 (25%)	2 (10%)		
Clear Cell	12 (7%)	3 (5%)	0 (0%)	1 (6%)		
Other	6 (3%)	7 (10%)	0 (0%)	0 (0%)		
Mixed	8 (4%)	2 (3%)	0 (0%)	2 (10%)		
**Pathology Grade**						
Low	24 (16%)	14 (23%)	1 (33%)	1 (6%)	0.550	0.550
Med	39 (26%)	14 (23%)	0 (0%)	4 (22%)		
High	86 (58%)	33 (54%)	2 (67%)	13 (72%)		
**Pathology Stage**						
1	77 (44%)	21 (34%)	1 (25%)	2 (11%)	**0.03**	0.15
2	34 (19%)	6 (10%)	0 (0%)	3 (16%)		
3	60 (34%)	32 (51%)	3 (75%)	13 (68%)		
4	6 (3%)	3 (5%)	0(0%)	1 (5%)		
**Residual Tumour post-surgery**						
None	123 (73%)	37 (63%)	1 (25%)	9 (56%)	**0.01**	0.06
<1cm	17 (10%)	6 (10%)	0 (0%)	4 (25%)		
1-2cm	7 (4%)	4 (7%)	0 (0%)	2 (13%)		

>2cm	22 (13%)	12 (20%)	3 (75%)	1 (6%)		
**Platinum Sensitivity**					0.333	0.333
Sensitive	150 (93%)	48 (87%)	3 (75%)	16 (94%)		
Resistant	11 (7%)	7 (13%)	1 (25%)	1 (6%)		

Supplementary Table S10: Nuclear Cyclin D1 expression and clinicopathological parameters.

**Parameters**	**Nuclear Cyclin D1 expression**			
	Low H score ≤180	***High H score >180***	***p value***	***Adjusted p value***
	N (%)	***N (%)***		
**Pathology Type**				
Serous	140 (57%)	0 (0%)	**0.001**	**0.005**
Mucinous	27 (11%)	4 (44%)		
Endometriod	31 (13%)	4 (44%)		
Clear Cell	19 (8%)	1 (12%)		
Other	14 (6%)	0 (0%)		
Mixed	13 (5%)	0 (0%)		
**Pathology Grade**				
Low	32 (15%)	0 (0%)	**<0.0001**	**<0.0001**
Med	37 (18%)	5 (83%)		
High	137 (67%)	1 (17%)		
**Pathology Stage**				
1	91 (39%)	7 (78%)	0.127	0.40
2	35 (15%)	0 (0%)		
3	100 (43%)	2 (22%)		
4	7 (3%)	0 (0%)		
**Residual tumour post-surgery**				
None	153 (69%)	7 (88%)	0.309	0.309
<1cm	28 (13%)	0 (0%)		
1-2cm	10 (4%)	1 (12%)		
>2cm	32 (14%)	0 (0%)		

**Platinum Sensitivity**			0.315	0.315
Sensitive	186 (90%)	9 (100%)		
Resistant	21 (10%)	0 (0%)		

Significant p values are in bold.

Adjusted p value was calculated using Bonferroni correction.

Supplementary Table S11: Cytoplasmic Cyclin D1 expression and clinicopathological parameters.

**Parameters**	**Cytoplasmic Cyclin D1 expression**		*p value*	*Adjusted p value*
	Low H score ≤10	*High H score>10*		
	N (%)	*N (%)*		
**Pathology Type**				
Serous	129 (56%)	11 (51%)	0.988	0.988
Mucinous	28 (12%)	3 (14%)		
Endometriod	31 (13%)	4 (18%)		
Clear Cell	18 (8%)	2 (9%)		
Other	13 (6%)	1 (4%)		
Mixed	12 (5%)	1 (4%)		
**Pathology Grade**				
Low	28 (14%)	4 (22%)	0.668	0.668
Med	39 (20%)	3 (17%)		
High	127 (66%)	11 (61%)		
**Pathology Stage**				
1	86 (39%)	12 (54%)	0.475	0.475
2	32 (15%)	3 (14%)		
3	95 (43%)	7 (32%)		
4	7 (3%)	0 (0%)		
**Residual Tumour Following Surgery**				
None	143 (68%)	17 (85%)	0.112	0.448
<1cm	27 (13%)	1 (5%)		
1-2cm	9 (4%)	2 (10%)		
>2cm	32 (15%)	0 (0%)		
**Platinum Sensitivity**			0.974	0.974
Sensitive	176 (90%)	19 (90%)		
Resistant	19 (10%)	2 (10%)		

Significant p values are in bold

Adjusted p value was calculated using Bonferroni correction.

Supplementary Table S12: Nuclear and cytoplasmic Cyclin D1 co-expression and clinicopathological parameters.

**Parameters**	**Cyclin D1 Nuclear (n) and cytoplasmic (c) co-expression**				*p value*	*Adjusted P value*
	Low (n)-low (c)	*High (n)-low (c)*	*High (n)-high (c)*	*Low (n)-high (c)*		
	N (%)	*N (%)*				
**Pathology Type**						
Serous	129 (57%)	0 (0%)	0 (0%)	11 (58%)	0.073	0.3500
Mucinous	25 (11%)	3 (50%)	1 (33%)	2 (10%)		
Endometriod	29 (13%)	2 (33%)	2 (67%)	2 (10%)		
Clear Cell	17 (8%)	1 (17%)	0 (0%)	2 (10%)		
Other	13 (6%)	0 (0%)	0 (0%)	1 (6%)		
Mixed	12 (5%)	0 (0%)	0 (0%)	1 (6%)		
**Pathology Grade**						
Low	28 (15%)	0 (0%)	0 (0%)	4 (25%)	**0.005**	**0.0350**
Med	36 (19%)	3 (75%)	2 (100%)	1 (6%)		
High	126 (66%)	1 (25%)	0 (0%)	11 (69%)		
**Pathology Stage**						
1	82 (38%)	4 (67%)	3 (100%)	9 (47%)	0.537	0.537
2	32 (15%)	0 (0%)	0 (0%)	3 (16%)		
3	93 (44%)	2 (33%)	0 (0%)	7 (37%)		
4	7 (3%)	0 (0%)	0(0%)	0 (0%)		
**Residual tumour posy-surgery**						
None	139 (68%)	4 (80%)	3 (100%)	14 (82%)	0.260	0.26
<1cm	27 (13%)	0 (0%)	0 (0%)	1 (6%)		
1-2cm	8 (4%)	1 (20%)	0 (0%)	2 (12%)		

>2cm	32 (15%)	0 (0%)	0 (0%)	0 (0%)		
**Platinum Sensitivity**					0.793	0.793
Sensitive	170 (90%)	6 (100%)	3 (100%)	16 (89%)		
Resistant	19 (10%)	0 (0%)	0 (0%)	2 (11%)		

Significant p values are in bold.

Adjusted p value was calculated using Bonferroni correction.

Supplementary Table S13. Nuclear Cyclin E1 expression and clinicopathological parameters.

**Parameters**	**Nuclear Cyclin E expression**			
	Low (*H score* ≤*140)*	*High* (*H score* >*140)*	***p value***	***Adjusted p value***
	N (%)	***N (%)***		
**Pathology Type**				
**Serous**	116 (58%)	34 (64%)	**0.031**	0.155
**Mucinous**	25 (13%)	3 (6%)		
**Endometriod**	31 (15%)	3 (6%)		
**Clear Cell**	12 (6%)	7 (13%)		
**Other**	11 (5%)	1 (2%)		
**Mixed**	7 (3%)	5 (9%)		
**Pathology Grade**				
**Low**	24 (14%)	7 (16%)	0.411	0.82
**Med**	38 (22%)	6 (13%)		
**High**	108 (64%)	32 (71%)		
**Pathology Stage**				
**1**	80 (42%)	15 (28%)	0.247	0.74

**2**	29 (15%)	8 (15%)		
**3**	78 (41%)	27 (51%)		
**4**	5 (2%)	3 (6%)		
**Residual tumour post Surgery**				
**Non**	134 (74%)	25 (50%)	**0.009**	0.06
**<1cm**	21 (12%)	9 (18%)		
**1-2cm**	8 (4%)	3 (6%)		
**>2cm**	19 (10%)	13 (26%)		
**Platinum Sensitivity**				
**Sensitive**	158 (93%)	39 (87%)	0.177	0.71
**Resistant**	12 (7%)	6 (13%)		

Supplementary Table S14: Cytoplasmic Cyclin E1 expression and clinicopathological parameters.

**Parameters**	**Cytoplasmic Cyclin E expression**		*p value*	*Adjusted P value*
	Low ≤80	***High>80***		
	N (%)	***N (%)***		
**Pathology Type**				
Serous	29 (37%)	121 (68%)	**<0.0001**	**<0.0001**
Mucinous	17 (22%)	11 (6%)		
Endometriod	13 (16%)	21 (12%)		
Clear Cell	11 (14%)	8 (5%)		
Other	4 (5%)	8 (5%)		
Mixed	5 (6%)	7 (4%)		
**Pathology Grade**				
Low	15 (24%)	16 (11%)	**0.041**	0.124
Med	12 (19%)	32 (21%)		
High	36 (57%)	104 (68%)		
**Pathology Stage**				
1	38 (50%)	57 (34%)	**0.031**	0.123
2	14 (18%)	23 (14%)		
3	24 (31%)	81 (48%)		
4	1 (1%)	7 (4%)		
**Residual Tumour post-surgery**				
None	59 (80%)	100 (63%)	0.090	0.18
<1cm	7 (9%)	23 (15%)		
1-2cm	2 (3%)	9 (6%)		
>2cm	6 (8%)	26 (16%)		
**Platinum Sensitivity**			0.871	0.817
Sensitive	62 (91%)	135 (92%)		
Resistant	6 (9%)	12 (8%)		

Significant p values are in bold.

Adjusted p value was calculated using Bonferroni correction.

Supplementary Table S15: Nuclear and cytoplasmic Cyclin E1 co-expression and clinicopathological parameters.

**Parameters**	**Nuclear (n) and cytoplasmic (c) co-expression**				*p value*	*Adjusted P value*
	Low (n)-low (c)	*High (n)-low (c)*	*High (n)-high (c)*	*Low (n)-high (c)*		
	N (%)	*N (%)*				
**Pathology Type**						
Serous	25 (37%)	4 (37%)	30 (73%)	91 (67%)	**0.001**	**0.006**
Mucinous	15 (22%)	2 (18%)	1 (2%)	10 (7%)		
Endometriod	12 (18%)	1 (9%)	2 (5%)	19 (14%)		
Clear Cell	8 (12%)	2 (18%)	4 (10%)	5 (4%)		
Other	4 (6%)	0 (0%)	1 (2%)	7 (5%)		
Mixed	3 (5%)	2 (18%)	3 (8%)	4 (3%)		
**Pathology Grade**						
Low	11 (21%)	4 (44%)	3 (9%)	13 (11%)	0.062	0.242
Med	11 (21%)	1 (12%)	5 (14%)	27 (23%)		
High	31 (58%)	4 (44%)	27 (77%)	78 (66%)		
**Pathology Stage**						
1	32 (49%)	6 (55%)	9 (22%)	48 (38%)	0.088	0.264
2	13 (20%)	1 (9%)	7 (17%)	16 (12%)		
3	19 (29%)	4 (36%)	22 (54%)	60 (47%)		
4	1 (2%)	0 (0%)	3 (7%)	4 (3%)		
**Residual tumour post-surgery**						
None	51 (81%)	7 (70%)	17 (44%)	84 (70%)	**0.013**	0.06
<1cm	7 (11%)	0 (0%)	9 (23%)	14 (12%)		

1-2cm	2 (3%)	0 (0%)	3 (8%)	6 (5%)		
>2cm	3 (5%)	3 (30%)	10 (25%)	16 (13%)		
**Platinum Sensitivity**					0.464	0.464
Sensitive	52 (91%)	9 (90%)	29 (85%)	107 (94%)		
Resistant	5 (9%)	1 (10%)	5 (15%)	7 (6%)		

Significant p values are in bold.

Adjusted p value was calculated using Bonferroni correction

Supplementary Table S16: Nuclear RB1 expression and clinicopathological parameters.

Parameters	Nuclear RB1 expression		*p value*	*Adjusted p value*
	Low	*High*		
	N (%)	*N (%)*		
**Pathology Type**				
Serous	98 (62%)	63 (49%)	0.134	0.40
Mucinous	20 (13%)	18 (14%)		
Endometriod	19 (12%)	19 (15%)		
Clear Cell	7 (4%)	16 (13%)		
Other	5 (3%)	5 (4%)		
Mixed	9 (6%)	7 (5%)		
**Pathology Grade**				
Low	21 (16%)	14 (13%)	0.736	0.736
Med	29 (21%)	21 (19%)		
High	86 (63%)	74 (68%)		
**Pathology Stage**				
1	59 (39%)	46 (37%)	0.817	0.817
2	21 (14%)	22 (18%)		
3	68 (45%)	52 (42%)		
4	4 (2%)	4 (3%)		
**Residual tumour post-surgery**				
None	102 (69%)	77 (69%)	0.599	0.599
<1cm	19 (13%)	13 (12%)		
1-2cm	9 (6%)	4 (3%)		
>2cm	17 (12%)	18 (16%)		

**Platinum Sensitivity**			**0.01**	0.06
Sensitive	128 (95%)	93 (86%)		
Resistant	7 (5%)	15 (14%)		

Significant p values are in bold.

Adjusted p value was calculated using Bonferroni correction.

Supplementary Table S17: Cytoplasmic RB1 expression and clinicopathological parameters.

**Parameters**	**Cytoplasmic RB1 expression**		*p value*	*Adjusted p value*
	Low	*High*		
	N (%)	*N (%)*		
**Pathology Type**				
Serous	136 (54%)	25 (72%)	0.304	0.618
Mucinous	36 (14%)	2 (6%)		
Endometriod	35 (14%)	3 (8%)		
Clear Cell	22 (9%)	1 (3%)		
Other	9 (4%)	1 (3%)		
Mixed	13 (5%)	3 (8%)		
**Pathology Grade**				
Low	33 (15%)	2 (7%)	0.138	0.690
Med	47 (22%)	3 (11%)		
High	137 (63%)	23 (82%)		
**Pathology Stage**				
1	94 (39%)	11 (32%)	0.196	0.784
2	40 (17%)	3 (9%)		
3	100 (41%)	20 (59%)		
4	8 (3%)	0 (0%)		
**Residual tumour post-surgery**				
None	160 (71%)	19 (58%)	**0.04**	0.2
<1cm	23 (10%)	9 (27%)		
1-2cm	12 (5%)	1 (3%)		

>2cm	31 (14%)	4 (12%)		
**Platinum Sensitivity**			0.282	0.84
Sensitive	194 (90%)	27 (96%)		
Resistant	21 (10%)	1 (4%)		

Significant p values are in bold.

Adjusted p value was calculated using Bonferroni correction.

Supplementary Table S18: Nuclear and cytoplasmic RB1 co-expression and clinicopathological parameters.

Parameters	RB1 Nuclear (n) and cytoplasmic (c) co-expression				*p value*	*Adjusted p value*
	Negative nuclear and cytoplasmic	*Positive nuclear, negative cytoplasmic*	*Positive nuclear and cytoplasmic*	*Positive cytoplasmic, negative nuclear*		
	N (%)	*N (%)*	*N (%)*	*N (%)*		
**Surgical Pathology Type**						
**Serous**	74 (60%)	62 (49%)	1 (100%)	24 (70%)	0.619	0.619
**Mucinous**	18 (14%)	18 (14%)	0 (0%)	2 (6%)		
**Endometriod**	16 (13%)	19 (15%)	0 (0%)	3 (9%)		
**Clear Cell**	6 (5%)	16 (13%)	0 (0%)	1 (3%)		
**Other**	4 (3%)	5 (4%)	0 (0%)	1 (3%)		
**Mixed**	6 (5%)	7 (5%)	0 (0%)	3 (9%)		
**Surgical Pathology Grade**						
**Low**	19 (17%)	14 (13%)	0 (0%)	2 (7%)	0.06	0.246
**Med**	27 (25%)	20 (18%)	1 (100%)	2 (7%)		
**High**	63 (58%)	74 (69%)	0 (0%)	23 (86%)		
**Surgical Pathology Stage**						
**1**	48 (41%)	46 (37%)	0 (0%)	11 (33%)	0.753	0.735
**2**	18 (15%)	22 (18%)	0 (0%)	3 (9%)		
**3**	49 (41%)	51 (42%)	1 (100%)	19 (58%)		
**4**	4 (3%)	4 (3%)	0(0%)	0 (0%)		
**Surgical Pathology Substage**						
**A**	24 (27%)	25 (25%)		3 (11%)	0.308	0.924
**B**	9 (10%)	12 (12%)		6 (23%)		

**C**	57 (63%)	63 (63%)		17 (65%)		
**Residual Tumour Following Surgery**						
**Non**	84 (73%)	76 (68%)	1 (100%)	18 (56%)	0.226	0.45
**<1cm**	10 (9%)	13 (12%)	0 (0%)	9 (28%)		
**1-2cm**	8 (7%)	4 (4%)	0 (0%)	1 (3%)		
**>2cm**	13 (11%)	18 (16%)	0 (0%)	4 (13%)		
**Platinum Sensitivity**					0.119	0.35
**Sensitive**	102 (94%)	92 (86%)	1 (100%)	26 (96%)		
**Resistant**	6 (6%)	15 (14%)	0 (0%)	1 (4%)		

Supplementary Table S19: Nuclear pRB1 expression and clinicopathological parameters.

**Parameters**	**Nuclear pRB1 expression**		*p value*	*Adjusted p value*
	Low	*High*		
	N (%)	*N (%)*		
**Pathology Type**				
Serous	137 (55%)	24 (70%)	0.380	0.380
Mucinous	35 (14%)	1 (3%)		
Endometriod	37 (15%)	3 (9%)		
Clear Cell	19 (8%)	2 (6%)		
Other	9 (3%)	2 (6%)		
Mixed	14 (5%)	2 (6%)		
**Pathology Grade**				
Low	32 (15%)	4 (13%)	0.850	0.850
Med	43 (20%)	5 (17%)		
High	138 (65%)	21 (70%)		
**Pathology Stage**				
1	97(40%)	8 (24%)	0.181	0.360
2	39(16%)	4 (12%)		
3	100 (41%)	19 (58%)		
4	7 (3%)	2 (6%)		
**Residual Tumour post-surgery**				
None	159 (70%)	19 (61%)	0.363	0.363
<1cm	26 (11%)	7 (23%)		
1-2cm	12 (5%)	1 (3%)		
>2cm	31 (14%)	4 (13%)		
**Platinum Sensitivity**			0.629	0.629
Sensitive	200 (91%)	23 (88%)		
Resistant	19 (9%)	3 (12%)		

Significant p values are in bold.

Adjusted p value was calculated using Bonferroni correction.

Supplementary Table S20: Cytoplasmic pRB1 expression and clinicopathological parameters.

Parameters	Cytoplasmic pRB1 expression		*p value*	*Adjusted p value*
	Low	*High*		
	N (%)	*N (%)*		
**Surgical Pathology Type**				
**Serous**	136 (54.6%)	25 (67.6%)	0.497	0.497
**Mucinous**	34 (13.7%)	2 (5.4%)		
**Endometriod**	34 (13.7%)	6 (16.2%)		
**Clear Cell**	21 (8.4%)	1 (2.7%)		
**Other**	10 (4%)	1 (2.7%)		
**Mixed**	14 (5.6%)	2 (5.4%)		
**Surgical Pathology Grade**				
**Low**	31 (14.8%)	5 (15.2%)	0.969	0.969
**Med**	41 (19.5%)	7 (21.2%)		
**High**	138 (65.7%)	21 (63.6%)		
**Surgical Pathology Stage**				
**1**	90 (37.7%)	15 (40.5%)	0.980	0.980
**2**	37 (15.5%)	6 (16.2%)		
**3**	104 (43.5%)	15 (40.5%)		
**4**	8 (3.3%)	1 (2.7%)		
**Residual Tumour Following Surgery**				
**Non**	153 (68%)	25 (73.5%)	0.512	0.512
**<1cm**	28 (12.4%)	5 (14.7%)		
**1-2cm**	13 (5.8%)	0 (0%)		
**>2cm**	31 (13.8%)	4 (11.8%)		
**Platinum Sensitivity**			0.199	0.39
**Sensitive**	191 (90.1%)	32 (97%)		
**Resistant**	21 (9.9%)	1 (3%)		

Significant p values are in bold.

Adjusted p value was calculated using Bonferroni correction.

Supplementary Table S21: Nuclear and cytoplasmic pRB1 co-expression and clinicopathological parameters

Parameters	Nuclear (n) and cytoplasmic (c) co-expression				*p value*	*Adjusted p value*
	Low (n)-low (c)	*High (n)-low (c)*	*High (n)-high (c)*	*Low (n)-high (c)*		
	N (%)	*N (%)*	*N (%)*	*N (%)*		
**Pathology Type**						
Serous	117 (53%)	20 (70%)	5 (62%)	19 (71%)	0.413	0.413
Mucinous	34 (15%)	1 (3%)	1 (13%)	0 (0%)		
Endometriod	33 (14%)	4 (14%)	2 (25%)	1 (4%)		
Clear Cell	18 (8%)	1 (3%)	0 (0%)	3 (11%)		
Other	8 (4%)	1 (3%)	0 (0%)	2 (7%)		
Mixed	12 (6%)	2 (7%)	0 (0%)	2 (7%)		
**Pathology Grade**						
Low	28 (15%)	4 (15%)	1 (17%)	3 (12%)	0.924	0.924
Med	38 (20%)	5 (18%)	2 (33%)	3 (12%)		
High	120 (65%)	18 (67%)	3 (50%)	18 (76%)		
**Pathology Stage**						
1	87 (41%)	10 (35%)	5 (63%)	3 (12%)	0.156	0.312
2	34 (16%)	5 (17%)	1 (12%)	3 (12%)		
3	87 (41%)	13 (45%)	2 (25%)	17 (68%)		
4	6 (2%)	1 (3%)	0 (0%)	2 (8%)		
**Residual Tumour post-surgery**						
None	138 (69%)	21 (78%)	4 (57%)	15 (63%)	0.364	0.364
<1cm	24 (12%)	2 (7%)	3 (43%)	4 (16%)		
1-2cm	12 (6%)	0 (0%)	0 (0%)	1 (5%)		

>2cm	27 (13%)	4 (15%)	0 (0%)	4 (16%)		
**Platinum Sensitivity**					0.451	0.451
Sensitive	175 (91%)	25 (96%)	7 (100%)	16 (84%)		
Resistant	18 (9%)	1 (4%)	0 (0%)	3 (16%)		

Significant p values are in bold.

Adjusted p value was calculated using Bonferroni correction.

Supplementary Figure 1

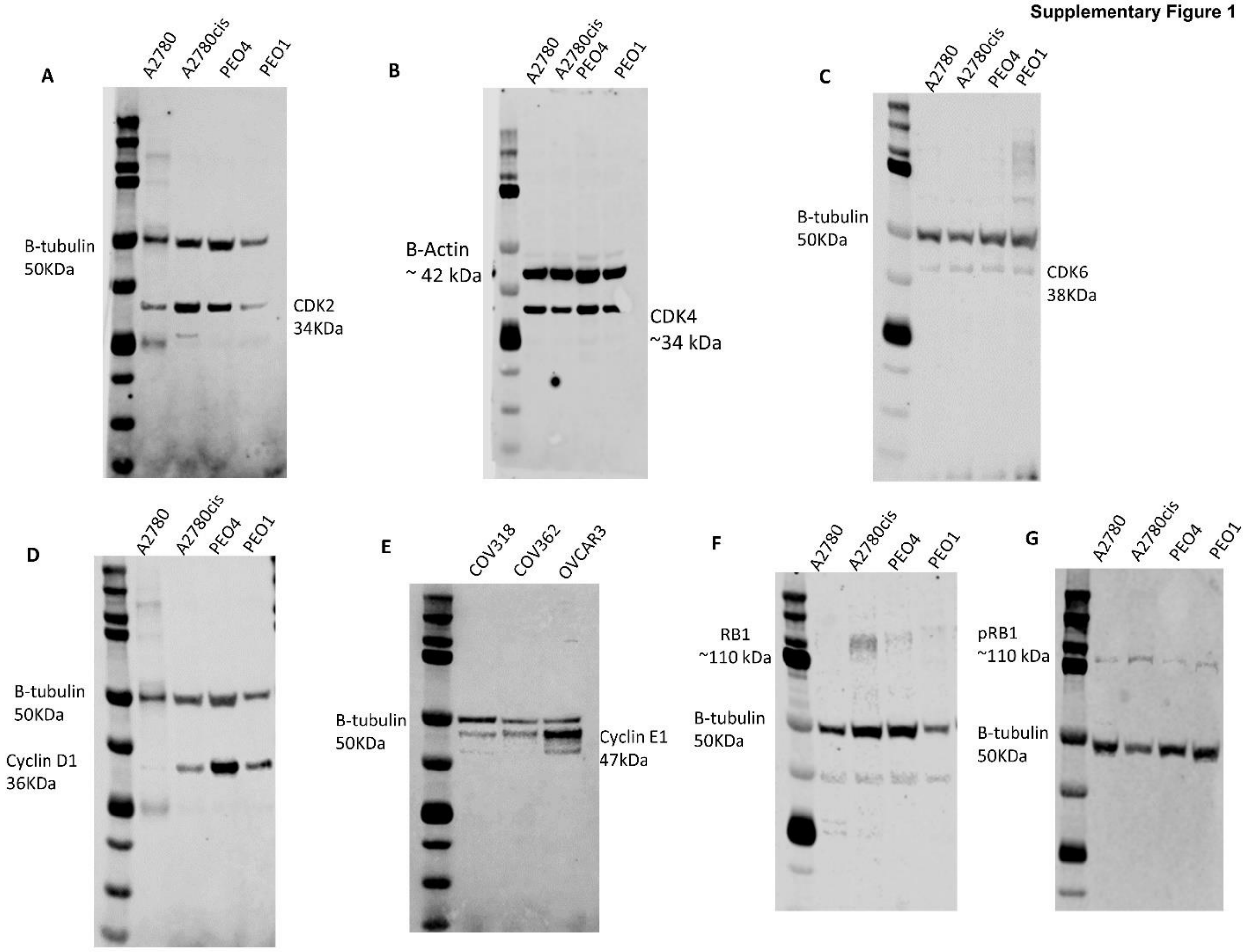

Supplementary Figure S1. A. Western blot showing CDK2 expression in PEO1, PRO4, A2780 and A2780cis ovarian cancer cell lines. **B.** Western blot showing CDK4 expression in PEO1, PRO4, A2780 and A2780cis ovarian cancer cell lines. **C.** Western blot showing CDK6 expression in PEO1, PRO4, A2780 and A2780cis ovarian cancer cell lines. **D.** Western blot showing cyclin D1 expression in PEO1, PRO4, A2780 and A2780cis ovarian cancer cell lines. **E.** Western blot showing cyclin E1 expression in PEO1, PRO4, A2780 and A2780cis ovarian cancer cell lines. **F.** Western blot showing RB1 expression in PEO1, PRO4, A2780 and A2780cis ovarian cancer cell lines. **G.** Western blot showing pRB1 expression in PEO1, PRO4, A2780 and A2780cis ovarian cancer cell lines.

Supplementary Figure 2

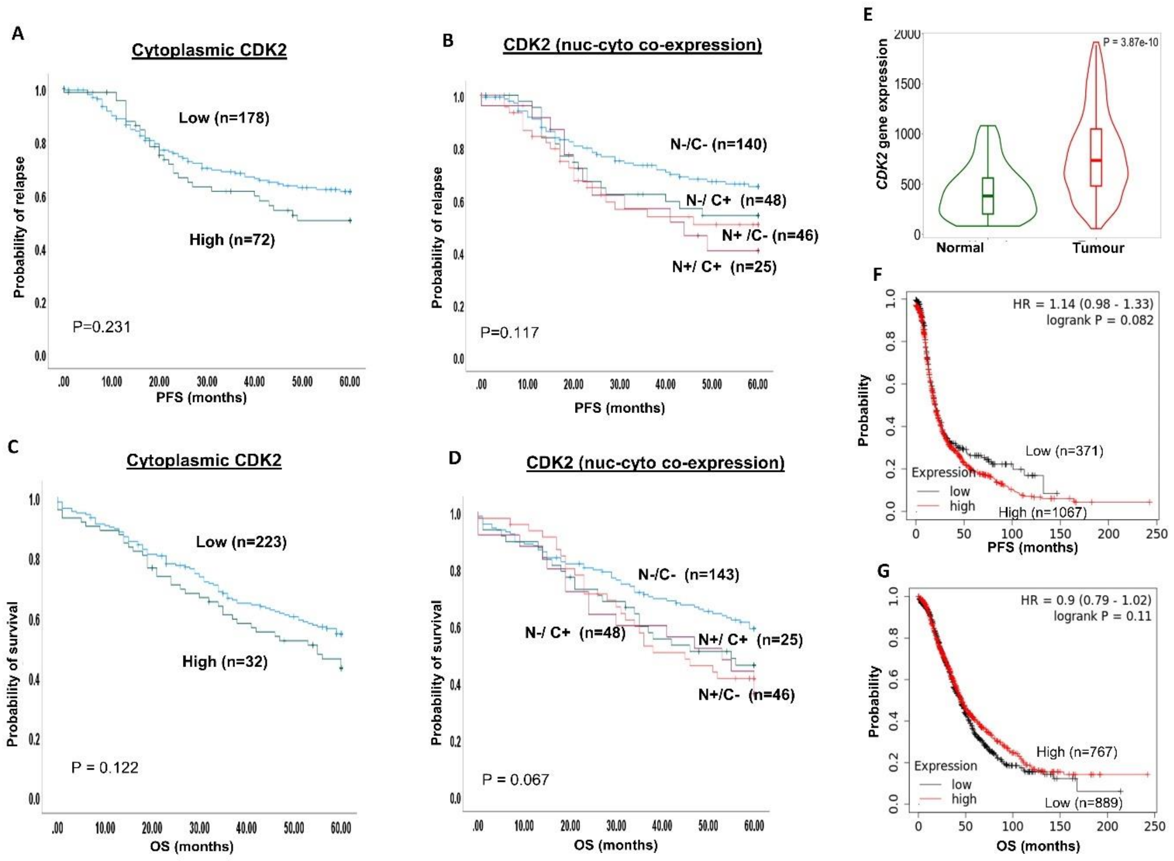

Supplementary Figure S2. A. Kaplan-Meier curve for CDK2 cytoplasmic expression and progression free survival. **B.** Kaplan-Meier curve for CDK2 nuclear &cytoplasmic co-expression and progression free survival. **C.** Kaplan-Meier curve for CDK2 cytoplasmic expression and overall survival. **D.** Kaplan-Meier curve for CDK2 nuclear &cytoplasmic co-expression and overall survival. **E.** Violin plot shows the median of *CDK2* mRNA expression in normal and ovarian tumors; the red and green central bars represent the median, long green, and red lines represent the interquartile range. On each side of the red and green lines is a kernel density estimation to show the distribution shape of the data. **F.** Kaplan-Meier curve for *CDK2* mRNA expression and progression free survival. **G.** Kaplan-Meier curve for *CDK2* mRNA expression and overall survival.

Supplementary Figure 3

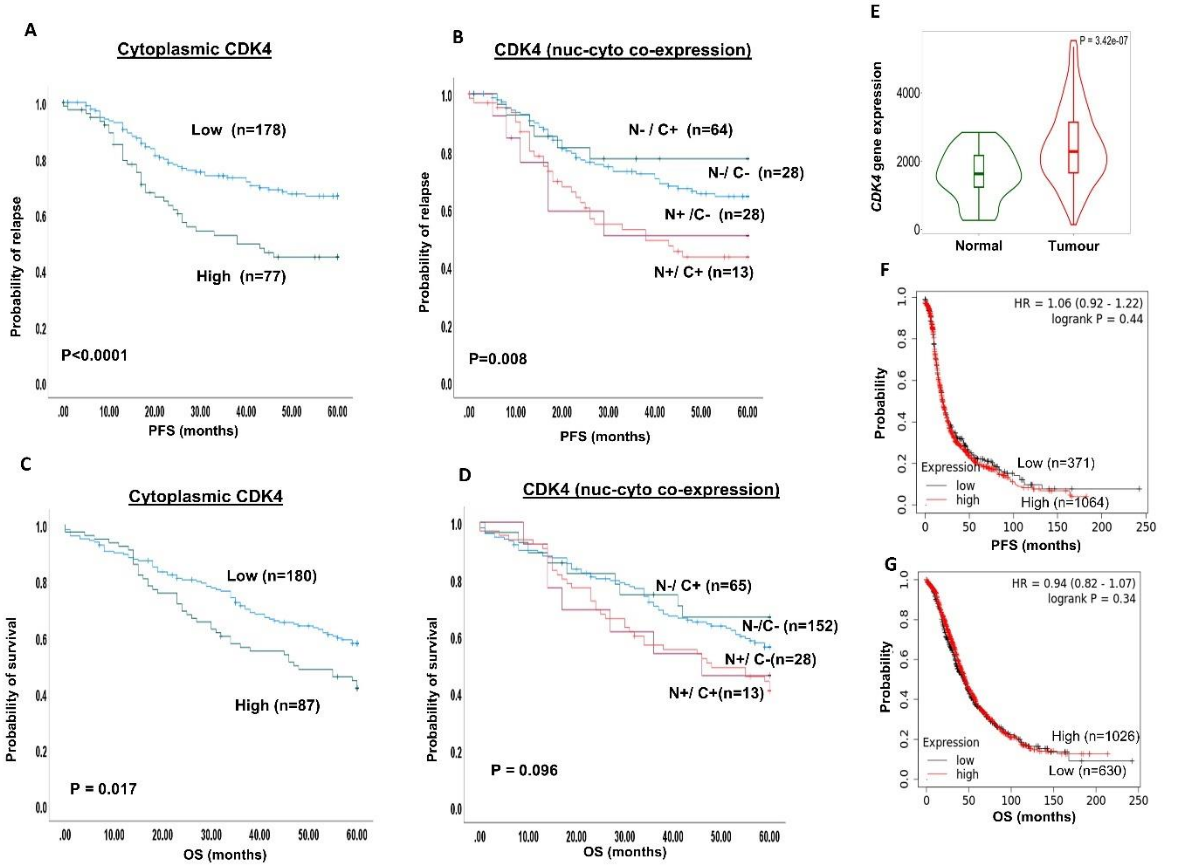

Supplementary Figure S3. A. Kaplan-Meier curve for CDK4 cytoplasmic expression and progression free survival. **B.** Kaplan-Meier curve for CDK4 nuclear &cytoplasmic co-expression and progression free survival. **C.** Kaplan-Meier curve for CDK4 cytoplasmic expression and overall survival. **D.** Kaplan-Meier curve for CDK4 nuclear &cytoplasmic co-expression and overall survival. **E.** Violin plot shows the median of *CDK4* mRNA expression in normal and ovarian tumors; the red and green central bars represent the median, long green, and red lines represent the interquartile range. On each side of the red and green lines is a kernel density estimation to show the distribution shape of the data. **F.** Kaplan-Meier curve for *CDK4* mRNA expression and progression free survival. **G.** Kaplan-Meier curve for *CDK4* mRNA expression and overall survival.

Supplementary Figure 4

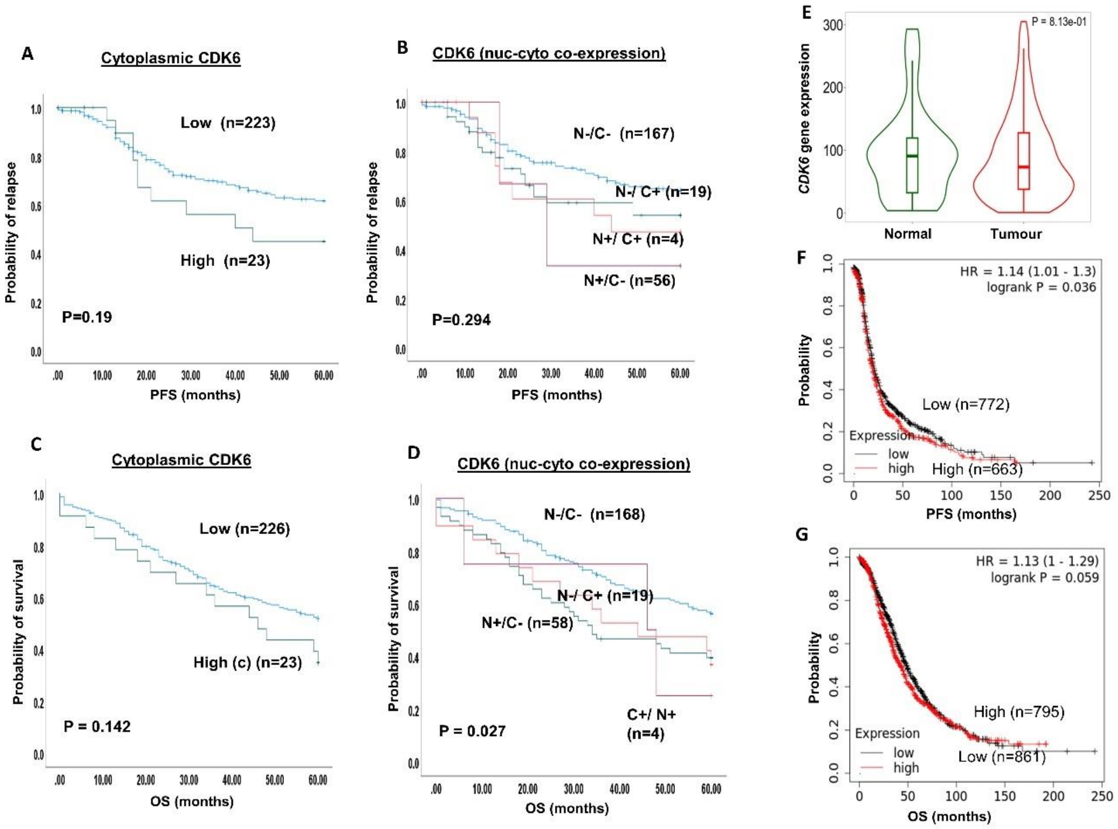

Supplementary Figure S4. A. Kaplan-Meier curve for CDK6 cytoplasmic expression and progression free survival. **B.** Kaplan-Meier curve for CDK6 nuclear &cytoplasmic co-expression and progression free survival. **C.** Kaplan-Meier curve for CDK6 cytoplasmic expression and overall survival. **D.** Kaplan-Meier curve for CDK6 nuclear &cytoplasmic co-expression and overall survival. **E.** Violin plot shows the median of *CDK6* mRNA expression in normal and ovarian tumors; the red and green central bars represent the median, long green, and red lines represent the interquartile range. On each side of the red and green lines is a kernel density estimation to show the distribution shape of the data. **F.** Kaplan-Meier curve for *CDK6* mRNA expression and progression free survival. **G.** Kaplan-Meier curve for *CDK6* mRNA expression and overall survival.

Supplementary Figure 5

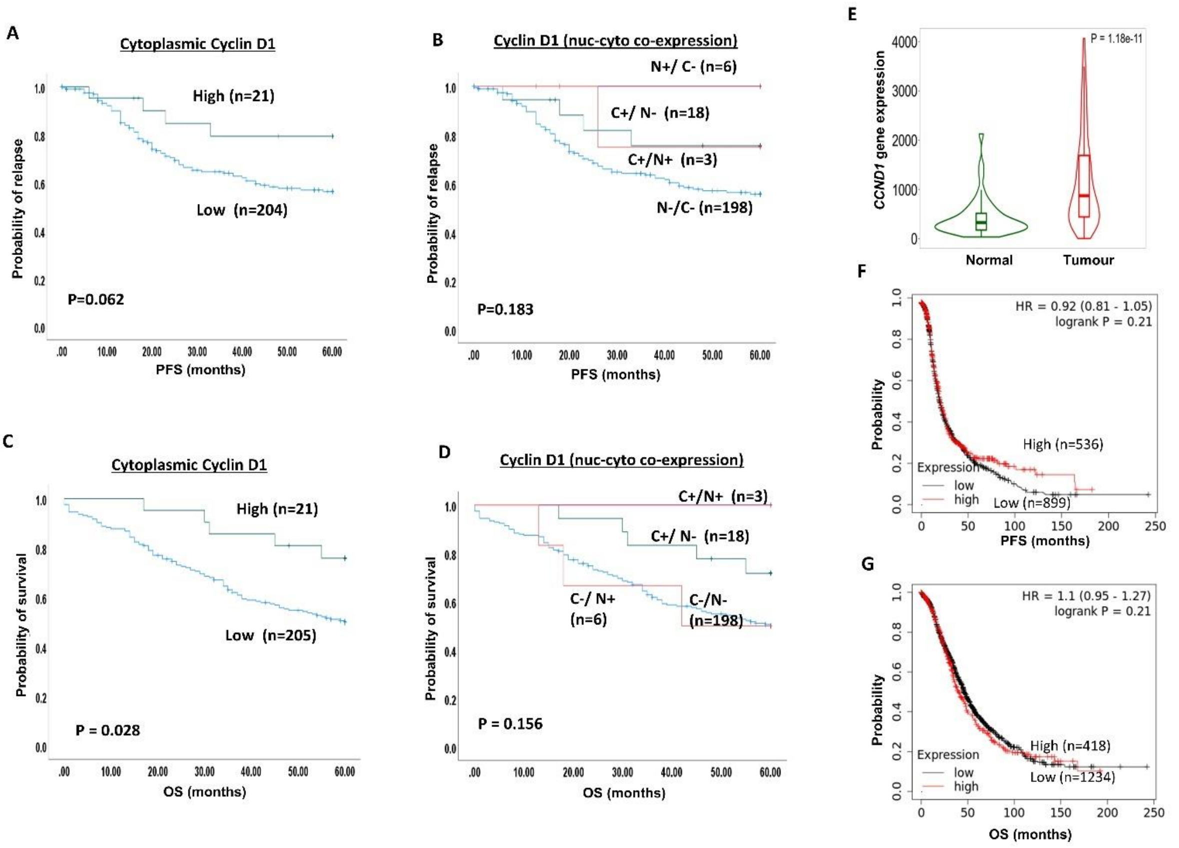

Supplementary Figure S5. A. Kaplan-Meier curve for Cyclin D1 cytoplasmic expression and progression free survival. **B.** Kaplan-Meier curve for Cyclin D1 nuclear &cytoplasmic co-expression and progression free survival. **C.** Kaplan-Meier curve for Cyclin D1 cytoplasmic expression and overall survival. **D.** Kaplan-Meier curve for Cyclin D1 nuclear &cytoplasmic co-expression and overall survival. **E.** Violin plot shows the median of *Cyclin D1* mRNA expression in normal and ovarian tumors; the red and green central bars represent the median, long green, and red lines represent the interquartile range. On each side of the red and green lines is a kernel density estimation to show the distribution shape of the data. **F.** Kaplan-Meier curve for *Cyclin D1* mRNA expression and progression free survival. **G.** Kaplan-Meier curve for *Cyclin D1* mRNA expression and overall survival.

Supplementary Figure 6

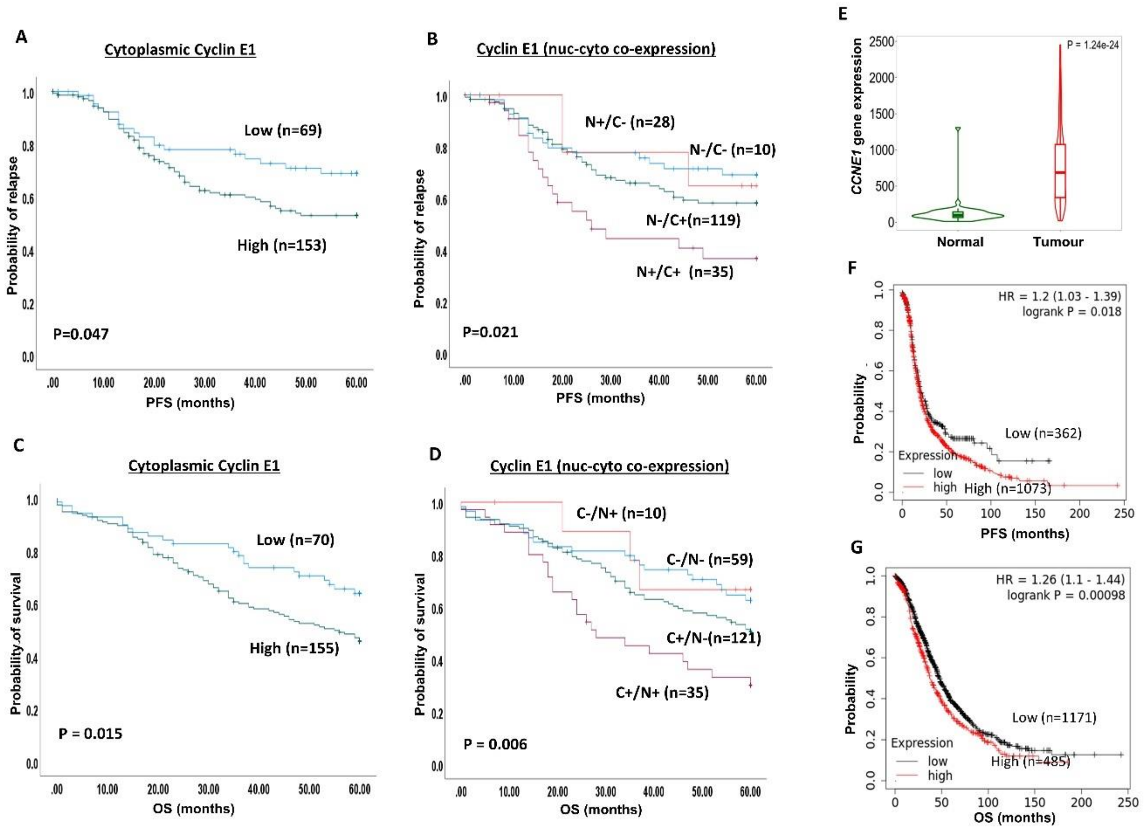

Supplementary Figure S6. A. Kaplan-Meier curve for Cyclin E1 cytoplasmic expression and progression free survival. **B.** Kaplan-Meier curve for Cyclin E1 nuclear &cytoplasmic co-expression and progression free survival. **C.** Kaplan-Meier curve for Cyclin E1 cytoplasmic expression and overall survival. **D.** Kaplan-Meier curve for Cyclin E1 nuclear &cytoplasmic co-expression and overall survival. **E.** Violin plot shows the median of *Cyclin E1* mRNA expression in normal and ovarian tumors; the red and green central bars represent the median, long green, and red lines represent the interquartile range. On each side of the red and green lines is a kernel density estimation to show the distribution shape of the data. **F.** Kaplan-Meier curve for *Cyclin E1* mRNA expression and progression free survival. **G.** Kaplan-Meier curve for *Cyclin E1* mRNA expression and overall survival.

Supplementary Figure 7

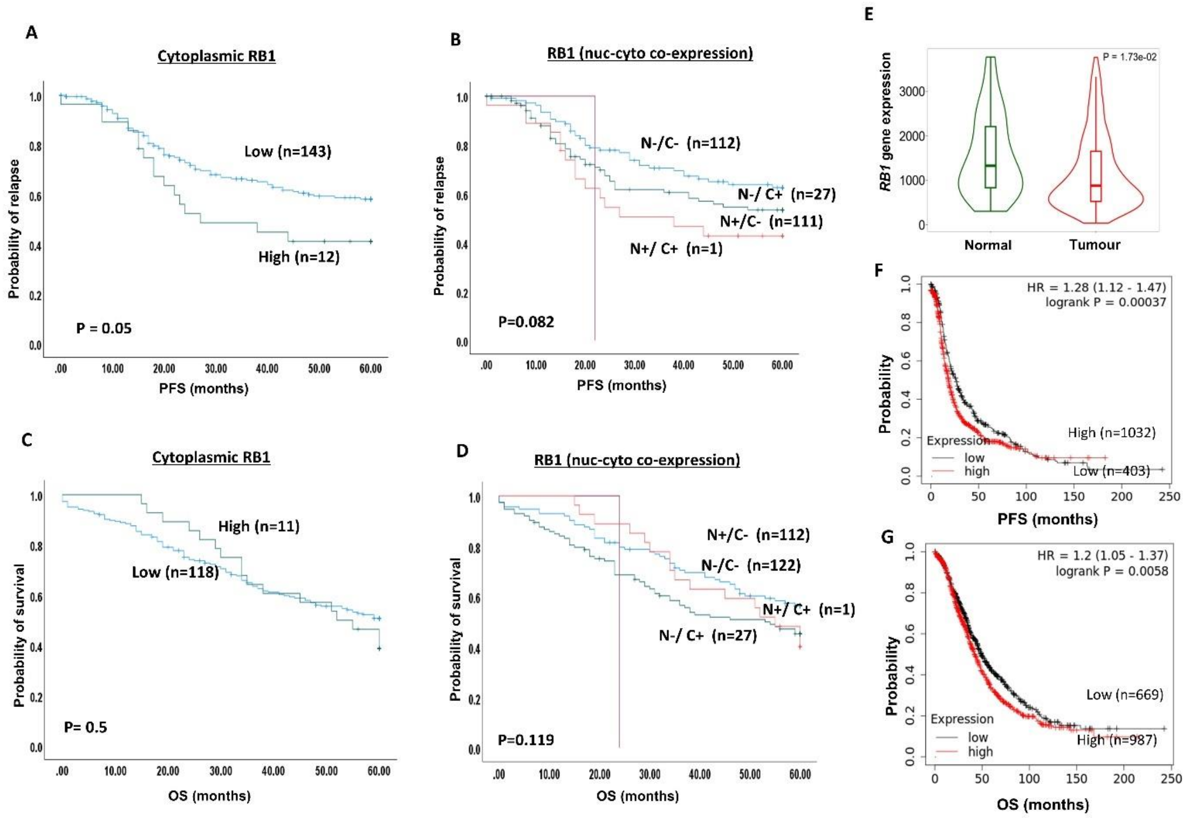

Supplementary Figure S7. A. Kaplan-Meier curve for RB1 cytoplasmic expression and progression free survival. **B.** Kaplan-Meier curve for RB1 nuclear &cytoplasmic co-expression and progression free survival. **C.** Kaplan-Meier curve for RB1 cytoplasmic expression and overall survival. **D.** Kaplan-Meier curve for RB1 nuclear &cytoplasmic co-expression and overall survival. **E.** Violin plot shows the median of *RB1* mRNA expression in normal and ovarian tumors; the red and green central bars represent the median, long green, and red lines represent the interquartile range. On each side of the red and green lines is a kernel density estimation to show the distribution shape of the data. **F.** Kaplan-Meier curve for *RB1* mRNA expression and progression free survival. **G.** Kaplan-Meier curve for *RB1* mRNA expression and overall survival.

Supplementary Figure 8

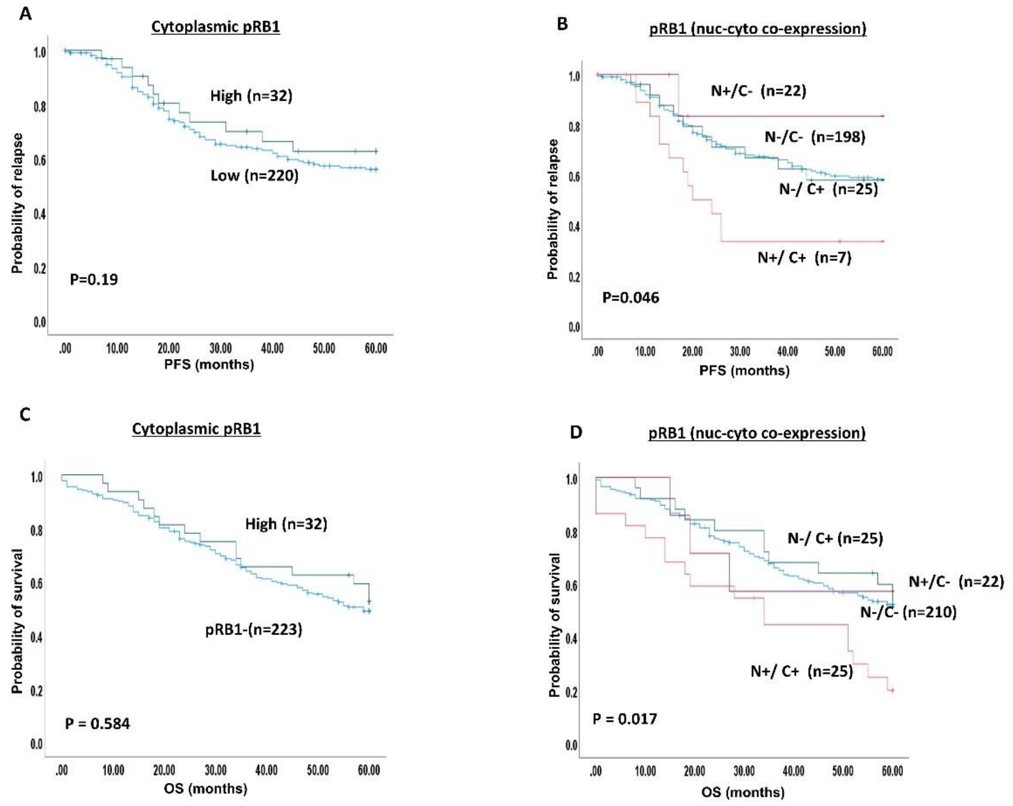

Supplementary Figure S8. A. Kaplan-Meier curve for pRB1 cytoplasmic expression and progression free survival. **B.** Kaplan-Meier curve for pRB1 nuclear &cytoplasmic co-expression and progression free survival. **C.** Kaplan-Meier curve for pRB1 cytoplasmic expression and overall survival. **D.** Kaplan-Meier curve for pRB1 nuclear &cytoplasmic co-expression and overall survival
